# Sustainable Carbon Aerogels from Polyolefin Plastics for High-Linearity Bidirectional Strain Sensing

**DOI:** 10.1007/s40820-026-02196-7

**Published:** 2026-05-09

**Authors:** Yang Yue, Hui Bi, Shiyu Zhang, Chen Luan, Zhangliu Tian, Dayong Ren, Fuqiang Huang

**Affiliations:** 1 State Key Laboratory of High-Performance Ceramics, Shanghai Institute of Ceramics, Chinese Academy of Sciences, Shanghai, 200050 People’s Republic of China; 2 Center of Materials Science and Optoelectronics Engineering, University of Chinese Academy of Sciences, Beijing, 100049 People’s Republic of China; 3 Zhejiang Key Laboratory of Industrial Solid Waste Thermal Hydrolysis Technology and Intelligent Equipment, Huzhou University, Huzhou, 313000 People’s Republic of China; 4 Key Laboratory of Intelligent Creation for Extreme Energy Materials of Ministry of Education, School of Materials Science and Engineering and Zhangjiang Institute for Advanced Study, Shanghai Jiao Tong University, Shanghai, 200240 People’s Republic of China

**Keywords:** Plastic upcycling, Sulfur-modulated catalysts, Hierarchical carbon aerogels, Coaxial bidirectional strain sensors, Linear and sensitive sensing region

## Abstract

Achieving highly linear and sensitive strain sensing under both tensile and compressive deformation remains a critical challenge in wearable electronics, as it demands a conductive network capable of reversible reconfiguration without compromising structural uniformity. This challenge is further intensified in hierarchical carbon nanostructures, where catalyst deactivation and unregulated carbon supply frequently lead to nonuniform nanocarbon growth and severely heterogeneous conductive pathways. Herein, we report a hierarchical carbon aerogel derived from plastics. Carbon nanofibers (CNFs) are in situ grown on elastic carbonized cotton fibers via plastic pyrolysis, enabled by Ni–S-modified catalytic interface and sustained carbon flux from plastic decomposition. The coupled regulation suppresses uneven nanocarbon deposition, yielding an elastic fibrous backbone densely interconnected by CNFs. The resulting network facilitates reversible reconstruction of conductive contacts under tension and compression, delivering a nearly linear electromechanical response over a broad bidirectional strain window with linear gauge factors of 7.8 at 82% tension and 1.7 at 28% compression, while maintaining stable sensitivity over 5000 cycles within a ± 20% strain window. Overall, this work achieves a wide bidirectional strain range, high sensitivity, and long-term stability, rarely combined in carbon-based strain sensors. Moreover, it reliably resolves strain direction and magnitude, enables sensitive adhesion sensing and joint-motion monitoring, highlighting its potential for next-generation human–machine interfaces.

**Supplementary Information:**

The online version contains supplementary material available at 10.1007/s40820-026-02196-7.

## Highlights

- Ni–S interfacial regulation combined with sustained carbon feeding from plastics enables uniform, controllable carbon nanofibers growth on cotton, producing a conductive and elastic carbon aerogel.
- Aerogel encapsulation delivers near-linear, wide-range bidirectional strain sensing without signal saturation or pronounced asymmetry.
- Coaxial bidirectional outputs quantify adhesion dynamics and extract a force-independent interfacial adhesion coefficient for bio-interfaces.

**Supplementary Information:**

The online version contains supplementary material available at 10.1007/s40820-026-02196-7.

## Graphical abstract

**Figure:**
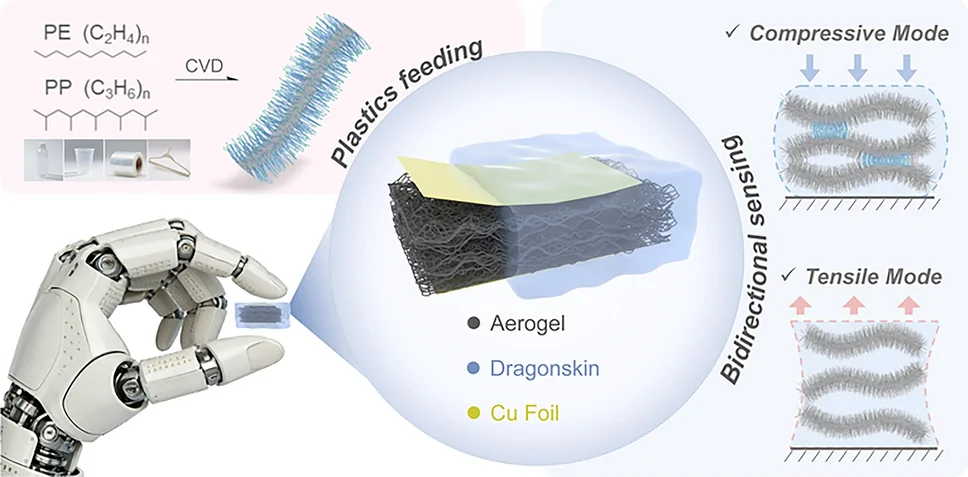

## Introduction

Flexible strain sensors translate mechanical deformation into electrical signals [1–3] and are central to emerging applications in medical monitoring [4–6], electronic skin [7–9], and soft robotics [10–13]. Recent advances in sensing-layer engineering have enabled tensile sensors capable of withstanding ultra-large strains (> 400%) [14–16] and compressive sensors operable under extreme compression (> 80%) [17, 18]. However, these advances have largely evolved along separate trajectories, with tensile and compressive sensing typically optimized independently rather than integrated within a single sensing layer. At a fundamental level, tensile and compressive deformations modulate electrical transport through distinct and often competing mechanisms. Under tensile strain, the separation of conductive fillers disrupts percolation pathways and weakens electrical connectivity [19]. In contrast, compressive deformation in porous or bioinspired architectures increases contact density and contact area, reinforcing conductive networks and amplifying resistance changes [20, 21]. This mechanistic asymmetry makes electrical decoupling of tension and compression intrinsically challenging, frequently leading to nonlinear, asymmetric, or ambiguous sensing responses [22]. Consequently, achieving a highly sensitive sensing layer capable of simultaneously resolving the direction and magnitude of coaxial bidirectional strain (tension and compression along the same axis) remains an unmet challenge.

Recent efforts have attempted to integrate tensile and compressive sensing within flexible devices. For example, Capasso et al*.* [23] incorporated 0D carbon nano-onions (CNOs) and 1D carbon nanotubes (CNTs) into a SEBS (styrene-ethylene-butylene-styrene) elastomer to formulate conductive inks, and fabricated stretchable (120%) and compressible (80%) sensors via PU-sponge impregnation. However, this system exhibited almost no linear working region. Beyond ~ 8% tensile strain, cracking of the conductive network induced pronounced signal fluctuations and severely compromised device stability and reliability. Gao et al*.* [24] assembled graphene and carbon nanotubes (CNTs) into a Kirigami-type stretch–compression carbon aerogel via 3D printing, achieving sensing from−14 to 100%. Nevertheless, the Kirigami geometry weakly perturbed conductive pathways under tension, resulting in a low tensile sensitivity even at large strain (GF = 0.1). These studies highlight the urgent need for nanomaterials that provide stable and reversible high-sensitivity windows while supporting coaxial bidirectional strain detection.

Natural cotton fibers are composed of twisted and entangled cellulose filaments with hollow interiors which give a high aspect ratio and decent elasticity (Fig. S1), making them attractive as three-dimensional scaffolds for multilevel sensing architectures [25, 26]. However, introducing secondary nanostructures onto carbonized cotton in a controlled manner remains challenging. Conventional transition-metal catalysts (Fe/Co/Ni) often undergo sintering or carbon encapsulation during growth [27, 28]. This leads to catalyst deactivation and nonuniform hierarchical structures that hinder predictable contact-conductance modulation and linear electromechanical responses (Fig. S2) [29, 30]. Thus, a new method that enables controllable growth and multilevel structural design is highly desirable for multimodal sensing layers.

**Fig. 1:**
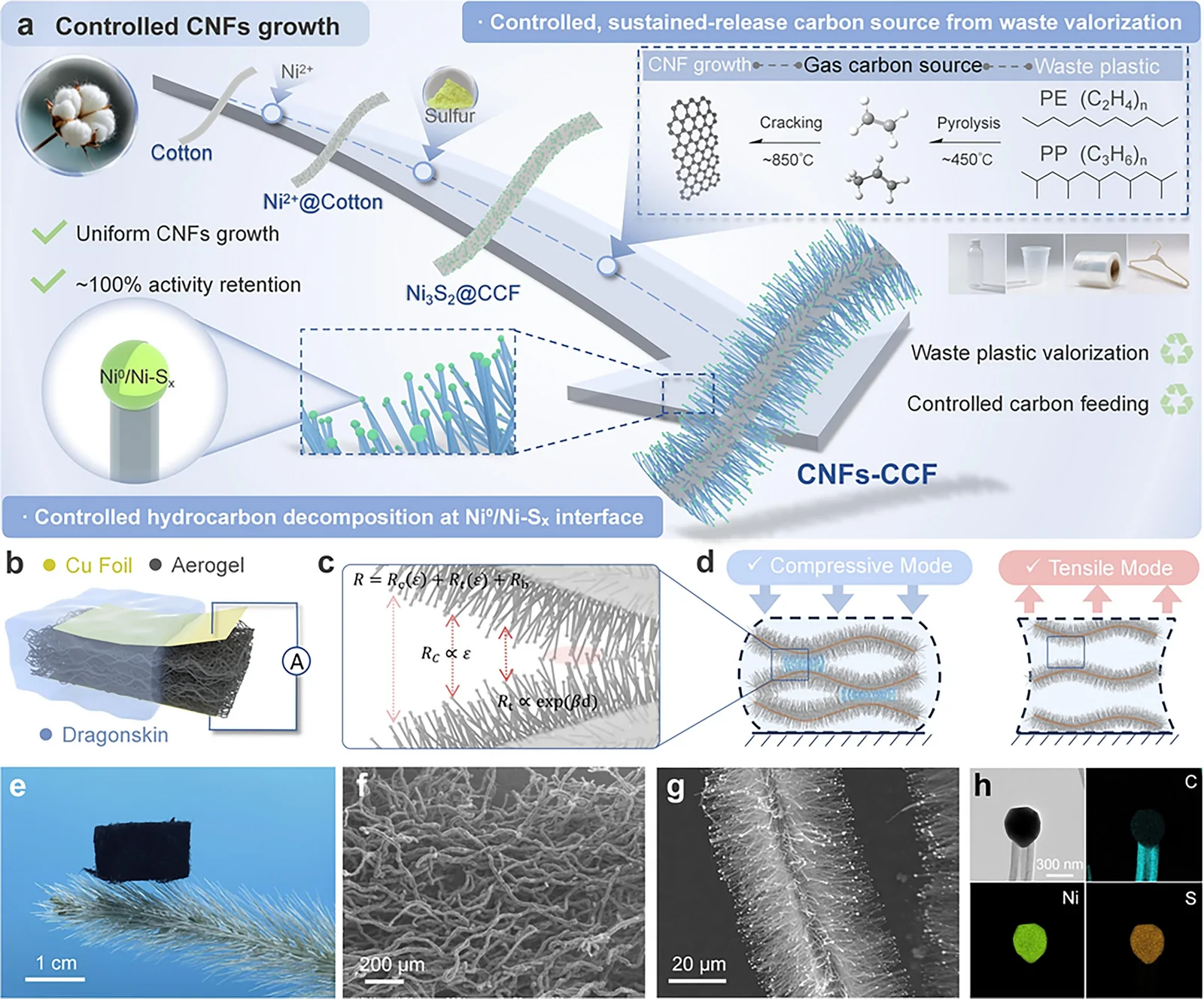
Schematic illustration of the controlled structural design of the CNFs-CCFs-A composite for a coaxial bidirectional strain sensor. **a** Schematic of the controlled growth of secondary CNFs on CCF using Ni^0^/Ni-S_*x*_ as the catalyst and polyolefin plastics (PP/PE) as the carbon source. **b** Structural composition of the CNFs-CCFs-A/Dragonskin composite sensing unit. **c** Schematic of the equivalent variation mechanisms of contact resistance (R_c_) and tunneling resistance (R_t_) during strain. **d** Schematic illustration of the macroscopic mechanical deformation and microscopic electrical variation of the sensing unit under tensile and compressive modes. **e** Optical photograph of the CNFs-CCFs-A. **f** Scanning electron microscopy (SEM) image of the CNFs-CCFs-A surface. **g** High-magnification SEM image of a single CNFs-CCF surface. **h** Energy-dispersive spectroscopy (EDS) elemental mapping of a single CNF

In this work, to achieve robust, high-performance bidirectional strain sensing, a structurally uniform CNF-bridged network is required. Thus, we target uniform, controllable CNF growth on a 3D cotton-derived CCF scaffold by introducing sulfur modulation and plastic feedstocks. Natural cotton fibers are employed as a three-dimensional structural framework, while Ni-S_*x*_ acts as an interfacial regulated catalyst in which Ni is stabilized through Ni–S interactions. Polyolefin plastics (PP/PE) are used as a sustainable carbon source (Fig. 1). A one-step chemical vapor deposition (CVD) process enables the formation of a uniform hierarchical carbon architecture, in which carbon nanofibers (CNFs) with nearly identical lengths are grown in situ on carbonized cotton fibers (CCFs), forming a stretchable and compressible aerogel (CNFs-CCFs-A) (Fig. 1a). The highly elastic CCF network provides stable three-dimensional conductive pathways and linear mechanical resilience under both tensile and compressive deformation. The introduction of CNFs further generates additional and tunable contact-conductance modulation, leading to larger resistance variations and enhanced strain sensitivity. After elastomer encapsulation, the aerogel exhibits a broad deformation range (~ 120% tension and ~ 50% compression) and a nearly linear electromechanical response, with a linear gauge factor of 7.8 at 82% tensile strain and 1.7 at 28% compressive strain. The device also maintains stable performance over more than 5000 tensile-compressive cycles between ± 20% strain. The resulting sensor can reliably resolve both the direction and magnitude of applied strain, while simultaneously achieving a wide bidirectional strain range and high sensitivity. This result addresses the long-standing trade-off between bidirectional strain range and sensitivity that has constrained carbon-based strain sensors. When integrated into electronic Dragonskin elastomer, it enables precise motion tracking and adhesion-related surface recognition, highlighting its potential for advanced wearable sensing applications.

## Experimental Section

### Materials Synthesis

#### Preparation of Carbonized Cotton Fibers

Degreased cotton was purchased from Shandong Dingtai Information Technology Co., Ltd. and used directly as the precursor. The cotton was placed in a tubular furnace and carbonized at 850 °C for 2 h under an argon atmosphere (Ar, 100 sccm), with a heating rate of 10 °C min^−1^. After cooling naturally to room temperature, carbonized cotton fibers (CCF) were obtained.

#### Preparation of Ni-Exchanged Cotton Fibers

Degreased cotton (0.5 g) was immersed in 50 mL of 10 mM Ni (NO_3_)_2_/ethanol solution until the solution was completely absorbed to ensure sufficient Ni^2+^ loading. The sample was then dried at 60 °C for 12 h, yielding Ni-exchanged cotton fibers.

#### ***Synthesis of Ni-S***_***x***_***-Catalyzed Carbon Nanofiber Aerogel (Ni-S***_***x***_***-CNFs-CCFs-A)***

Ni-S_*x*_-CNFs-CCFs-A was synthesized in a dual-zone tube furnace (Fig. S44). Ni-exchanged cotton fibers were placed in the high-temperature zone, while sulfur powder (0.3 g) and polypropylene (PP, 4 g) were placed in the low-temperature zone. Both zones were heated simultaneously. The high-temperature zone was raised to 850 °C within 80 min at a heating rate of 10 °C min^−1^, while the low-temperature zone was heated to 300 °C. The low-temperature zone was then further increased to 450 °C to trigger PP pyrolysis and generate carbon species. The reaction was maintained for 2 h under an Ar/H_2_ (100/15 sccm) atmosphere. After cooling to room temperature, Ni-S_*x*_-CNFs-CCFs-A was obtained.

#### Synthesis of Ni-Catalyzed Carbon Nanofiber Aerogel (Ni-CNFs-CCFs-A)

Ni-CNFs-CCFs-A was synthesized following the same procedure as Ni-S_*x*_-CNFs-CCFs-A, except that sulfur powder was not added. All other experimental conditions were kept identical.

Unless otherwise specified, CNFs-CCFs-A refers to samples synthesized at 850 °C using Ni-S_*x*_ catalyst.

### Material Characterization

Surface morphologies were characterized using field-emission scanning electron microscopy (FE-SEM, Hitachi S-4800). High-resolution transmission electron microscopy (TEM) images were acquired on a JEOL 1400 microscope operated at 120 kV. Crystal structures were analyzed by X-ray diffraction (XRD, Bruker D8 Advance) using Cu Kα radiation (λ = 1.5406 Å) over a scanning range of 10–80°. Raman spectra were collected on a LabRAM HR Evolution spectrometer using a 532 nm laser. Specific surface area and pore structure were determined by N_2_ adsorption–desorption measurements using an ASAP 2460–4 system. Samples were degassed at 150 °C under vacuum for 12 h prior to measurement. Surface chemical states were analyzed by X-ray photoelectron spectroscopy (XPS, Thermo Scientific ESCALAB 250). Plastic pyrolysis products were analyzed using a gas chromatograph (Agilent 7890 GC) equipped with a thermal conductivity detector (TCD), two flame ionization detectors (FID), and a DB-5 ms capillary column.

### Device Fabrication (Fabrication of CNFs-CCFs-A/Dragonskin Strain Sensors)

The prepared carbon aerogel was cut into blocks with dimensions of 15 mm × 10 mm × 10 mm. Dragonskin silicone elastomer was prepared by mixing component A and component B at a mass ratio of 1:1. The carbon aerogel was placed into a mold (30 mm × 15 mm × 15 mm) and fully encapsulated with the pre-cured elastomer. The mold was then heated at 80 °C for 30 min to obtain CNFs-CCFs-A/Dragonskin strain sensors with fully cured silicone encapsulation.

### Device Measurements

The electromechanical performance of the strain sensors was evaluated using an electrochemical workstation (Autolab PGSTAT 302 N, Switzerland). Sensors were fixed along the length direction using 706# silicone adhesive on a SUST CMT1102 mechanical testing system equipped with a 50 N load cell. Tensile and compressive tests were conducted using programmed loading protocols. In the wearable demonstrations (Fig. 5f–i, later), the CNFs-CCFs-A/Dragonskin composite was attached to the skin using medical-grade double-sided tape, and the electrical leads were secured with the same tape to minimize motion artifacts at the contact points. For alignment, the sensor’s long axis was oriented along the primary skin-strain direction associated with joint flexion and extension, which is approximately aligned with the joint rotation axis for these motions. Prior to data acquisition, a brief calibration movement was performed to confirm consistent waveform responses.

### Density Functional Theory Calculations

Density functional theory (DFT) calculations were performed using the Vienna Ab initio Simulation Package (VASP). The exchange–correlation interactions were described using the generalized gradient approximation (GGA) with the Perdew-Burke-Ernzerhof (PBE) functional, and the projector augmented wave (PAW) method was employed. A plane-wave energy cutoff of 500 eV was used.

Slab models of Ni_3_S_2_ (020) and Ni (111) surfaces were constructed, with a 20 Å vacuum layer along the surface normal direction. The Brillouin zone was sampled using a 3 × 3 × 1 Monkhorst–Pack k-point mesh. Structural optimization was performed until the total energy converged to 10^–5^ eV, and the residual atomic forces were below 0.05 eV Å^−1^. C_2_ and C_3_ carbon species were adsorbed on the Ni_3_S_2_ (020) and Ni (111) surfaces for geometry optimization. The adsorption energy (E_ads_) was calculated according to:

$${E}_{\mathrm{ads}}={E}_{\mathrm{total}\left(\mathrm{surface}+\mathrm{molecule}\right)}-{E}_{\mathrm{surface}}-{E}_{\mathrm{molecule}}$$

## Results and Discussion

### Controlled Growth and Structural Design

As a carbon aerogel material, CNFs-CCFs-A is constructed using natural cotton fibers as a three-dimensional elastic scaffold, on which carbon nanofibers are grown in situ under the synergistic catalysis of Ni^0^/Ni-S_*x*_ to form a hair-like hierarchical nanostructure (Fig. 1a). This design integrates a compliant fibrous framework, a controllable carbon source, and a deactivation-resistant catalytic interface, enabling coordinated regulation of both material structure and growth kinetics. The abundant hydroxyl groups and intrinsic porosity of cotton fibers (Fig. S3) facilitate efficient adsorption of Ni^2+^ ions, which are converted during carbonization into uniformly distributed Ni_3_S_2_ nanoparticles anchored on the surface of CCFs. With increasing temperature, the cellulose backbone undergoes dehydroxylation and aromatization to form a continuous carbon network (Fig. S4), while in the presence of reducing carbon species metallic Ni^0^ active sites are gradually generated to drive the nucleation and growth of carbon nanofibers. This process preserves the macroscopic integrity of the cotton-derived scaffold while constructing a mechanically resilient and electrically conductive CCF framework. Polyolefin plastics are employed as a sustainable carbon source, which undergo staged cracking at ~ 450 °C and further pyrolysis at ~ 850 °C to continuously release gaseous carbon species (Fig. 1a, right). This process supplies a kinetically stable and uniform carbon flux that avoids local oversaturation, catalyst encapsulation, and deactivation. Compared with conventional metallic Ni catalysts (Fig. S2), the Ni-S_*x*_-modified interface, through strong Ni–S interactions, suppresses particle agglomeration and excessive carbon dissolution [31], thereby enabling moderated hydrocarbon decomposition and controlled carbon dissolution–precipitation behavior, which promotes the uniform growth of carbon nanofibers on the CCF surface to form a structurally consistent hierarchical carbon network while simultaneously enhancing catalytic stability (Fig. 1a, left).

Figure 1b, c highlights the hierarchical architecture of CNFs-CCFs-A in multilayer composite systems. Carbon nanofibers are uniformly distributed on the CCF surface, forming a setaria-like hierarchical structure. The electrical response of the nanofibrous aerogel is described by the combined contributions of contact resistance (R_c_), tunneling resistance (R_t_), and bulk resistance (R_b_) (Fig. 1c). At low strain, R_c_ changes nearly linearly with a relatively stable R_b_, giving good linearity. At larger deformation, tension mainly drives pathway redistribution and makes R_b_ more strain-dependent, whereas compression primarily reduces R_t_ via increased contact and shortened spacing, leading to higher sensitivity and eventual saturation at high compression. Moreover, owing to the naturally elastic cotton-derived scaffold, CNFs-CCFs-A encapsulated with Dragonskin elastomer exhibits excellent structural stability and reversible deformation under both compressive and tensile modes (Fig. 1d). This behavior is mainly attributed to the ultra-high aspect ratio and soft, porous structure of cotton fibers, which allow stress to be accommodated through elastic deformation rather than brittle fracture.

Further macro- and microstructural characterizations (Fig. 1e–g) clearly reveal the overall morphology and local structural features of CNFs-CCFs-A. The CCF scaffold displays a typical one-dimensional high-aspect-ratio structure with an average diameter of approximately 4 µm (Fig. 1f, g). Carbon nanofibers are homogeneously distributed over the entire fiber surface, with no obvious aggregation or localized carbon deposition, indicating excellent uniformity and scalability of the growth strategy. In contrast, Ni-CNFs-CCF exhibits evident catalyst encapsulation within the CNFs, leading to catalyst deactivation (Fig. S5). Elemental mapping results (Fig. 1h) further confirm the coexistence of Ni and S, providing direct evidence for the crucial role of the Ni-S_*x*_ active phase in carbon nanofiber growth.

The controllable growth of CNFs depends on the coordinated regulation of the Ni-S_*x*_ interface and the carbonization temperature, in which the Ni-S_*x*_ composition is a key factor for hierarchical structure formation during CVD. As shown in Fig. S6, Ni-S_*x*_ phases (including Ni_3_S_2_ and Ni_7_S_6_) are formed in situ on the cotton fibers after sulfur introduction. Upon subsequent carbon-source feeding, the emergence of a metallic Ni^0^ peak indicates partial reduction of Ni-S_*x*_ to catalytically active Ni during CNF growth. When excessive sulfur is added, the Ni^0^ progressively diminishes, suggesting limited reducibility of highly sulfurized Ni-S_*x*_ and consequent suppression of CNF growth. At a fixed Ni (NO_3_)_2_ loading of 10 mM, an optimal Ni–S ratio close to 1:1 establishes a dynamic equilibrium between Ni-S_*x*_ and Ni^0^, enabling uniform CNF growth on the cotton scaffold (Fig. S7). The carbonization temperature also affects CNF growth by regulating the carbon dissolution and precipitation behavior in the catalyst. At 700 °C, the cracking of the carbon source and reduction of Ni^0^ is insufficient, leading to a limited density of catalytically active sites and consequently short and thin CNFs. At 1000 °C, more complete reduction of Ni and rapid carbon-source decomposition promote fast carbon dissolution and precipitation, resulting in the formation of a continuous carbon layer on the cotton fibers and suppressing one-dimensional nanofiber growth (Figs. S8 and S9). In contrast, at 850 °C, a moderate carbon solubility and controlled precipitation favor carbon deposition on catalyst particles, and together with the secondary decomposition of ethylene on the sidewalls, uniform carbon nanofiber networks are formed. Consistent with this growth scenario, statistical analysis reveals that Ni-S_*x*_-derived CNFs exhibit a smaller average diameter and a longer average length than those grown on Ni, indicating more controlled growth under Ni-S_*x*_ regulation. This quantitative trend supports that sulfur introduction suppresses excessive radial growth while promoting sustained axial extension, thereby strengthening the interconnected fibrous network (Fig. S10 and Table S1).

The uniform growth of CNFs is mainly governed by two factors: the thermal decomposition behavior of the plastic carbon source and the carbon dissolution–precipitation capability of the catalyst. Polypropylene (PP) and polyethylene (PE) undergo radical-driven chain scission in the temperature range of 400–500 °C (Fig. S11). Thermogravimetric, infrared, and mass spectrometry analyses show that increasing the pyrolysis temperature significantly accelerates the decomposition rate and increases the fraction of small-molecule products, resulting in a higher gaseous carbon flux (Fig. S12). When PP pyrolysates generated at different temperatures are used as the carbon source for CVD growth, Ni catalysts exhibit excessive carbon uptake due to favorable energy-level matching with carbon and inherently high carbon solubility [30]. At 400 and 450 °C, long-chain waxy or tar-like species are readily captured on the Ni surface, forming carbonaceous droplets or gel-like layers that rapidly carbonize and lead to spherical carbon aggregation rather than one-dimensional growth [32]. At 500 °C, although small-molecule carbon species favor carbon dissolution and precipitation, the excessive carbon flux still causes rapid encapsulation and deactivation of the Ni catalyst. In contrast, the presence of stable Ni–S bonds weakens the Ni–C interaction and suppresses excessive carbon dissolution (Fig. S13) [31, 33, 34]. Notably, when a representative post-consumer black PP packaging box was used as the carbon feedstock under the same conditions, we still obtained a CNFs-CCFs morphology with a comparable CNF uniformity (Fig. S14), indicating that the Ni-S_*x*_-regulated growth remains effective for post-consumer waste plastics rather than being limited to polyolefin plastic. As a result, the catalyst maintains stable activity across different carbon sources and enables controlled carbon dissolution and precipitation at the surface. Consequently, uniform CNFs networks are consistently formed on Ni_3_S_2_ catalysts. Even under the high carbon flux generated at 500 °C, the effect is limited to a reduced CNFs density rather than catalyst deactivation.

### Ni-S Regulated CNF Growth

**Fig. 2:**
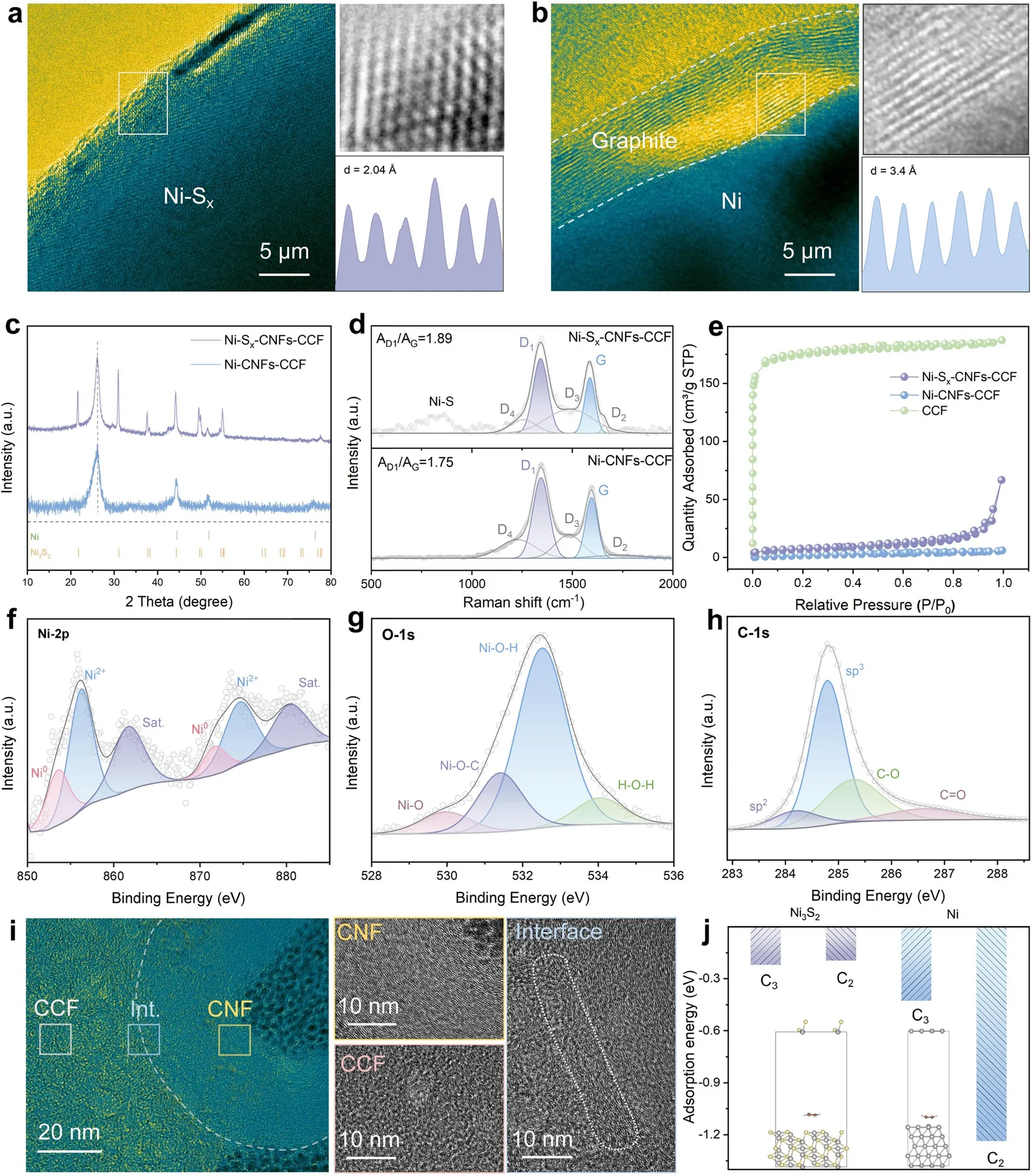
Microstructural and compositional characterization of the hierarchical CNFs-CCF regulated by the Ni-S_*x*_ catalytic interface. Transmission electron microscopy (TEM) images of CNF growth catalyzed by **a** Ni-S_*x*_ and **b** Ni, together with the corresponding high-resolution TEM (HRTEM) images of the selected regions. The insets show lattice fringe spacing analyses. **c** X-ray diffraction (XRD) patterns and **d** Raman spectra of Ni-S_*x*_-CNFs-CCF and Ni-CNFs-CCF. **e** Nitrogen adsorption–desorption isotherms of Ni-S_*x*_-CNFs-CCF, Ni-CNFs-CCF, and CCF. **f–h** X-ray photoelectron spectroscopy (XPS) results of Ni-S_*x*_-CNFs-CCF, including high-resolution spectra of Ni 2*p*, S 2*p*, and C 1*s*. **i** HRTEM image of the CNFs/CCF interface prepared by focused ion beam (FIB), revealing the structural features of carbonized cotton fibers (CCF), carbon nanofibers (CNFs), and their interface. **j** Calculated adsorption energies of carbon-source molecules on different interface models (Ni_3_S_2_-C_x_ and Ni-C_*x*_)

Figure 2 systematically illustrates the intrinsic differences between CNFs grown on Ni-S_*x*_ catalysts and metallic Ni catalysts in terms of structure, chemical states, and interfacial behavior, highlighting the critical role of sulfur in regulating carbon growth. As shown in Fig. 2a, b, HRTEM reveals pronounced interfacial differences. Carbon nanostructures grown on the Ni-S_*x*_ catalytic system show almost no continuous graphitic encapsulation on the catalyst surface, only localized graphite precipitates are observed at the Ni-S_*x*_–C interface. The corresponding electron diffraction patterns display lattice features of both Ni_3_S_2_ and Ni (Figs. S15 and S16) which is consistent with the weak Ni peaks observed in the XRD patterns. This result indicates that the introduction of sulfur significantly modulates carbon dissolution and diffusion at the Ni–C interface, thereby suppressing graphite encapsulation of the catalyst. The line-scan results show that the sulfur content remains nearly constant from the outer region to the interior of the catalyst particle, Ni exhibits slight enrichment in the central region, while carbon does not exhibit edge enrichment (Fig. S17). The high-angle annular dark-field scanning transmission electron microscopy (HAADF-STEM) elemental mapping further supports this conclusion (Fig. S18). In contrast, CNFs grown on Ni display well-ordered graphitic layers with an interlayer spacing of ~ 3.4 Å at the tip [35], suggesting catalyst encapsulation by graphite and a high likelihood of catalyst deactivation.

The XRD patterns (Fig. 2c) show that Ni-S*ₓ*-CNFs-CCF retains the characteristic diffraction peaks of Ni_3_S_2_ while also exhibiting Ni^0^ peaks arising from the formation of active metallic Ni under the reducing carbon source, whereas Ni-CNFs-CCF is dominated by graphitic carbon signals, indicating that metallic Ni^0^ plays a critical role in carbon precipitation and graphitization. Raman spectra (Figs. 2d and S19) show typical D and G bands for three samples. The slightly higher I_D1_/I_G_ ratio of Ni-S_*x*_-CNFs-CCF indicates an increased defect density, consistent with sulfur-induced structural distortion [35]. In addition, an extra Raman band in the 750–850 cm⁻^1^ range is observed for Ni-S_*x*_-CNFs-CCF, which can be attributed to Ni–S or Ni–C–S interfacial vibrational modes. This feature confirms the involvement of sulfur in regulating interfacial carbon deposition during CNFs growth [35]. Nitrogen adsorption–desorption measurements (Figs. 2e and S20) show that Ni-S_*x*_-CNFs-CCF possesses a more hierarchical pore structure, consistent with the formation of a uniformly distributed CNFs network and an open framework. XPS analysis (Figs. 2f-h and S21) further supports these observations. The Ni 2*p* and S 2*p* spectra reveal the presence of Ni–S bonds together with partially oxidized sulfur species. The C 1*s* spectrum contains dominant *sp*^2^ carbon along with minor C–O and C=O components (Fig. S22), suggesting interfacial defects and partial functionalization. Interfacial HRTEM images (Fig. 2i) show a continuous transition between CNFs, the interfacial layer, and the CCF substrate, which is favorable for stress transfer and structural stability [36]. Density functional theory calculations (Figs. 2j and S23) show that the adsorption energy of carbon species on Ni_3_S_2_ is significantly weaker than on metallic Ni. This result confirms that Ni–S bonding effectively weakens the Ni–C interaction, suppressing excessive carbon dissolution and catalyst poisoning. Overall, Ni_3_S_2_ regulates the electronic structure and adsorption behavior at the catalyst–carbon interface, enabling controlled carbon dissolution and precipitation [36]. This mechanism prevents graphite encapsulation and promotes uniform CNFs growth with stable hierarchical architectures.

### Mechanical Properties

**Fig. 3:**
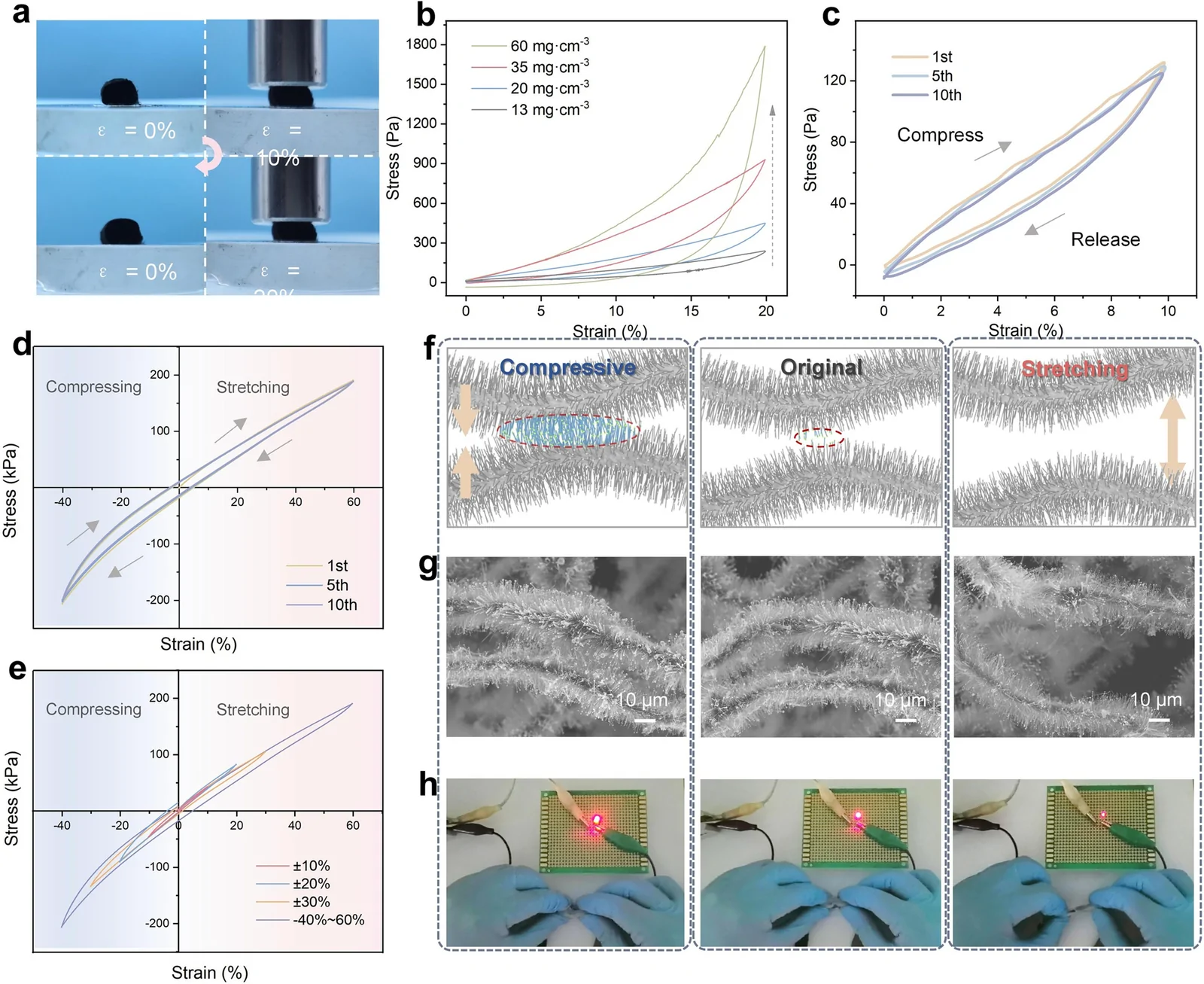
Mechanical performance of CNFs-CCFs-A and schematic illustration of the strain-sensing mechanism. **a** Optical image of CNFs-CCFs-A after recovery from 20% compressive strain. **b** Stress–strain curves of CNFs-CCFs-A with different densities. **c** Stress–strain curves of CNFs-CCFs-A with a density of 20 mg cm^−3^ during cyclic compression at 10% strain. **d** Cyclic compression (up to 40%) and stretching (up to 60%) tests of the CNFs-CCFs-A/Dragonskin flexible sensor. **e** Stress–strain curves of the flexible sensor under different strain ranges. **f** Schematic illustration of contact evolution within the CNFs-CCFs network during deformation and **g** corresponding high-resolution SEM images at different strain states, together with **h** photographs showing brightness changes of a connected light-emitting diode (LED)

Figure 3 presents the mechanical performance of CNFs-CCFs-A and its application as a flexible strain sensor. As shown in Figs. 3a and S24, CNFs-CCFs-A fully recovers its original shape after 20% compressive strain, demonstrating excellent elastic resilience. In contrast, the denser CNFs-CCFs-A shows difficulty in recovering after 20% strain (Fig. S25). The stress–strain curves of CNFs-CCFs-A with different densities (Fig. 3b) clearly exhibit hysteresis between compression and release, which arises from reversible structural rearrangement and energy dissipation within the porous network [37]. Meanwhile, conductivity data at different densities show that CNF introduction markedly boosts overall conductivity, indicating conductive heterogeneity between CNFs and CCFs (Fig. S26). Cyclic compression tests at 10% strain (Fig. 3c) show nearly overlapping stress–strain curves, indicating good mechanical stability and fatigue resistance under small deformations. Additionally, when the compressive strain amplitude is increased to 40%, the first loading–unloading cycle shows a small irreversible deformation, which can be attributed to the onset of network yielding and localized irreversible fracture of a few thicker CNF bundles. Importantly, the subsequent cycles become stable and highly reproducible with minimal further drift (Figs. S27 and S28). After integration with Dragonskin elastomer, the CNFs-CCFs-A/Dragonskin flexible sensor maintains stable mechanical responses under cyclic compression up to 40% and stretching up to 100% (Fig. 3d). The stress–strain curves remain continuous and distinguishable across different strain ranges (Fig. 3e), confirming the structural integrity of the composite sensor over a wide deformation window. Meanwhile, the introduction of sulfur also enhances the wettability between the CNFs-CCFs-A surface and the Dragonskin [14] (Fig. S29). The strain-sensing mechanism is illustrated in Fig. 3f. During compression and stretching, the number and area of contact points among CNFs change reversibly, and the significant difference in electrical conductivity between CCF and CNF (Fig. S30) leads to dynamic reconstruction of conductive pathways. High-resolution SEM images (Fig. 3g) directly capture the reversible evolution of the CNF network under different strain states. Furthermore, the LED demonstration (Fig. 3h) shows reversible brightness variations in response to mechanical deformation, confirming the efficient coupling between mechanical strain and electrical conductivity. Overall, the hierarchical porous structure and reversible contact modulation within the CNFs-CCFs-A network enable excellent elastic recovery, cycling stability, and reliable strain sensing over a broad strain range, highlighting its potential for flexible and wearable electronic applications.

### Electromechanical Sensing Performances

**Fig. 4:**
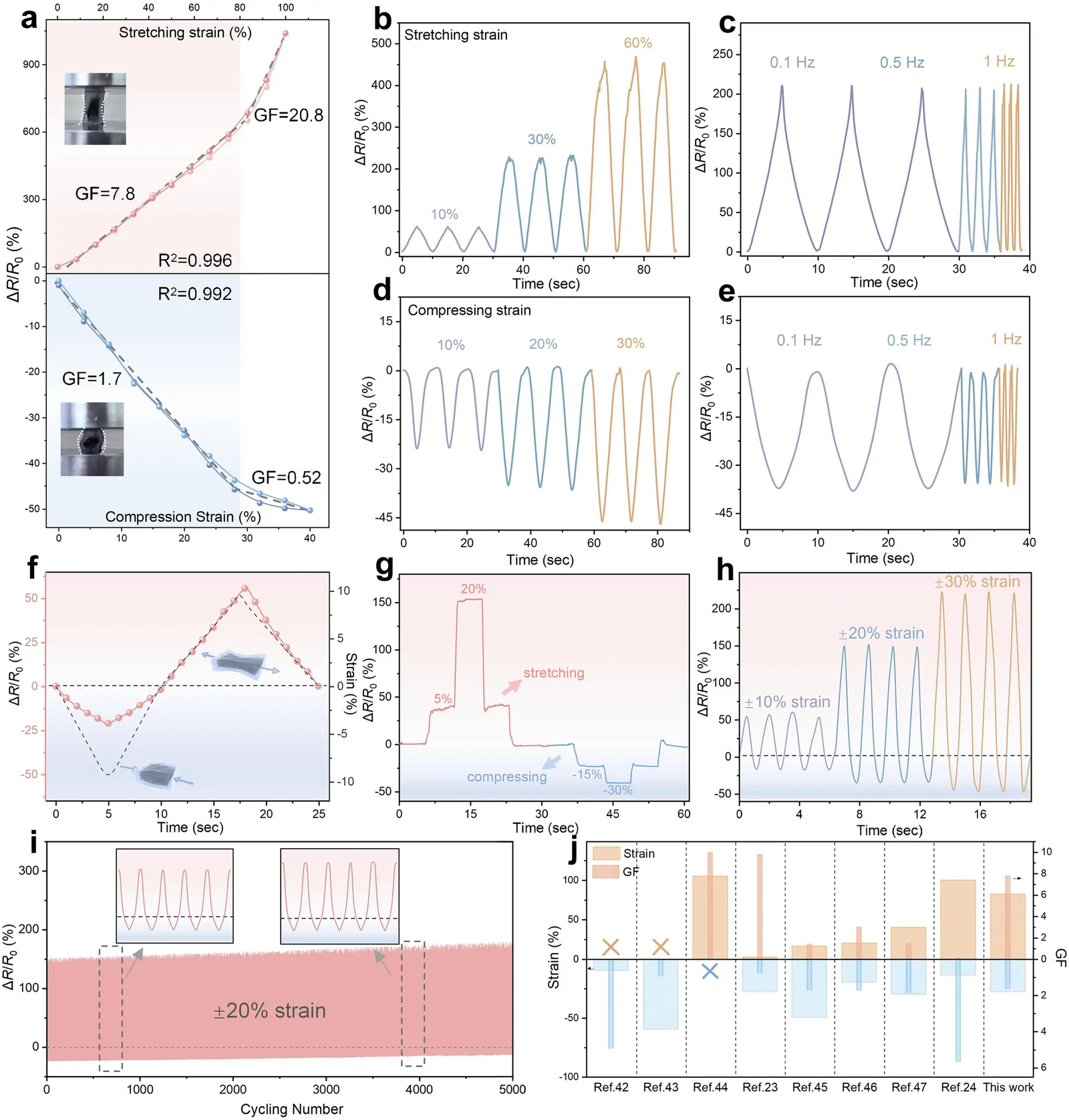
Electromechanical properties of the CNFs-CCFs-A/Dragonskin strain sensor. **a** Stretch–recovery response of the composite under tensile strains from 0 to 100% and compression–recovery response of the composite under compressive strains from 0 to 40%. The color-shaded background indicates the linear region. **b, c** Signal reliability and repeatability under cyclic tensile strain between 10% and 60% at a fixed frequency of 0.1 Hz, and at frequencies from 0.1 to 1 Hz under a fixed tensile strain of 30%. **d, e** Signal reliability and repeatability under cyclic compression between 10% and 30% at a fixed frequency of 0.1 Hz, and at frequencies from 0.1 to 1 Hz under a fixed compressive strain of 20%. **f** Continuous time–response showing the resistance change of the sensor under bidirectional strains from − 10% (compression) to + 10% (tension), where Δ*R* = *R* − *R*_0_. **g** Relative resistance variation (Δ*R*/*R*_0_) under sequential stepped strains from 0 to + 20% (tension), then to − 30% (compression), and finally returning to the initial state. **h** Signal reliability under alternating tensile and compressive strains between ± 10% and ± 30%. **i** Durability test over 5000 cycles between + 20% tensile and − 20% compressive strain. **j** Comparison of the linear tensile and compressive strain ranges and their corresponding gauge factors (GF, GF = (Δ*R*/*R*_0_)/*ε*, where *ε* is defined as the engineering strain (Δ*L*/*L*_0_)) of the CNFs-CCFs-A/Dragonskin sensor with those reported in previous studies

Figure 4 systematically presents the electromechanical responses of the CNFs-CCFs-A/Dragonskin composite strain sensor under compressive and tensile deformation. As shown in Figs. 4a and S31, the sensor remains responsive over a broad deformation window from 50% compression to 120% tension. Within − 40–100% strain, the resistance response is stable and highly reversible, as evidenced by the nearly symmetric loading–unloading curves and low hysteresis (2.22% for tensile cycling and 2.88% for compressive cycling). These results indicate that the conductive network can be controllably reconstructed during deformation and largely recovered upon release. To describe electromechanical linearity more rigorously, we performed linear regression and defined the linear region as the strain interval in which the fit satisfies R^2^ ≥ 0.99 and the normalized residuals show no systematic trend. Based on this criterion, the response exhibits quasi-linear behavior up to 82% in tension and 27% in compression (Fig. S32). The quasi-linearity within these ranges is primarily attributed to the uniformly grown CNF network, which stabilizes the percolation pathways while allowing conductive contacts to evolve progressively under deformation. Accordingly, the electrical signal originates from strain-dependent variations in the effective resistance pathways of the hierarchical conductive network, which can be expressed as:

$$R\left( \varepsilon \right) = R_{{\mathrm{b}}} + R_{{\mathrm{c}}} \left( \varepsilon \right) + R_{{\mathrm{t}}} \left( \varepsilon \right)$$

The electrical response of the nanofibrous aerogel is governed by contact resistance (R_c_) [38], tunneling resistance (R_t_) [39], and bulk resistance (R_b_) (Fig. 1c), whose relative contributions evolve differently under compression and tension. Under compression, network densification increases junction contact area and shortens inter-domain spacing, mainly reducing R_t_ and R_c_ and giving a fast response at low-moderate strain [40]. At higher compression, junction formation saturates and EIS shows enhanced junction-related capacitive effects (Figs. S33 and S34), consistent with the gradual saturation of Δ*R*/*R*_0_. Under tension, the difference between CCFs-A and CNFs-CCFs-A primarily arises from the introduction of CNFs as high-conductivity pathways. CNF bridges enhance connectivity and sensitivity at small-to-moderate strain, whereas at higher strain partial CNF disconnection drives current to redistribute toward the more continuous, lowly conductive CCF backbone (Fig. S35a). Given the > 2-orders-of-magnitude conductivity contrast (Fig. S30), this redistribution strengthens the strain dependence and promotes nonlinearity, occurring earlier at lower CNF density (Fig. S35b). Similarly, the CNFs-CCFs-A/Dragonskin device fabricated using a black plastic packaging box as the carbon feedstock exhibits comparably robust electrical responses, confirming the effectiveness of the Ni-S_*x*_-regulated growth with waste plastics (Fig. S36). In contrast, Ni-CNFs-CCFs-A/Dragonskin shows highly unstable signals over − 40–100% strain, indicating irreversible damage and pronounced rearrangement in its nonuniform network (Fig. S37).

Moreover, the silicone encapsulation (Poisson’s ratio ≈ 0.2) (Fig. S38) induces lateral contraction and multiaxial constraint [41], which amplifies the separation of critical conductive pathways and accounts for the pronounced asymmetry between tensile and compressive responses. The cotton framework is pre-stretched during assembly, yet SEM from the X/Y/Z directions shows the CCF scaffold remains overall quasi-random. X/Y/Z-directional measurements further confirm that Poisson-induced Y/Z deformation produces only weak transverse responses and does not noticeably distort the X-direction signal or its linearity within the working range (Fig. S39). Cyclic tests at different strain amplitudes (Fig. 4b, d) show good signal repeatability and stability within compressive strains of 10%−30% and tensile strains of 10–60%, with negligible signal drift. Frequency-dependent measurements (Fig. 4c, e) indicate that the resistance response accurately follows external strain in the frequency range of 0.1–1 Hz, confirming fast dynamic response and rapid signal recovery.

The electromechanical properties of the CNFs-CCFs-A/Dragonskin device were further examined by continuously applying tensile-compressive deformation in the coaxial direction. Continuous sinusoidal loading tests (Fig. 4f) further demonstrate reliable strain monitoring and consistent response under multiple deformation modes. After a step strain to 20%, the CNFs-CCFs-A/Dragonskin sensor exhibits a significant signal response, and it recovers to its initial state after relaxation. Upon subsequent step compression to−30%, the sensor exhibits a reversed signal, in agreement with the theoretical analysis (Fig. 4g). Subsequent continuous tension–compression electromechanical tests with different strain variables were performed in the same device. The sensor also shows a recognizable and reproducible correspondence with the stable resistance plateau, demonstrating the excellent creep resistance of the internal carbon aerogel (Fig. 4h). As the loading rate increases, the output signal remains stable and repeatable (Fig. S40). In addition, the sensor shows reliable mechanical durability even after 5000 cycles of 20% tension to 20% compression (Fig. 4i). The cyclic waveforms remain unchanged basically, demonstrating the mechanical structural integrity of the CNFs-CCFs-A/Dragonskin even under higher strain (Fig. S41). Table S2 and Fig. 4j show that the CNFs-CCFs-A/Dragonskin sensor exhibits a superior linear sensitive window during coaxial bidirectional sensing, while also delivering comparable performance under heteroaxial sensing conditions [23, 24, 42–47].

### Wearable Sensing Demonstrations

**Fig. 5:**
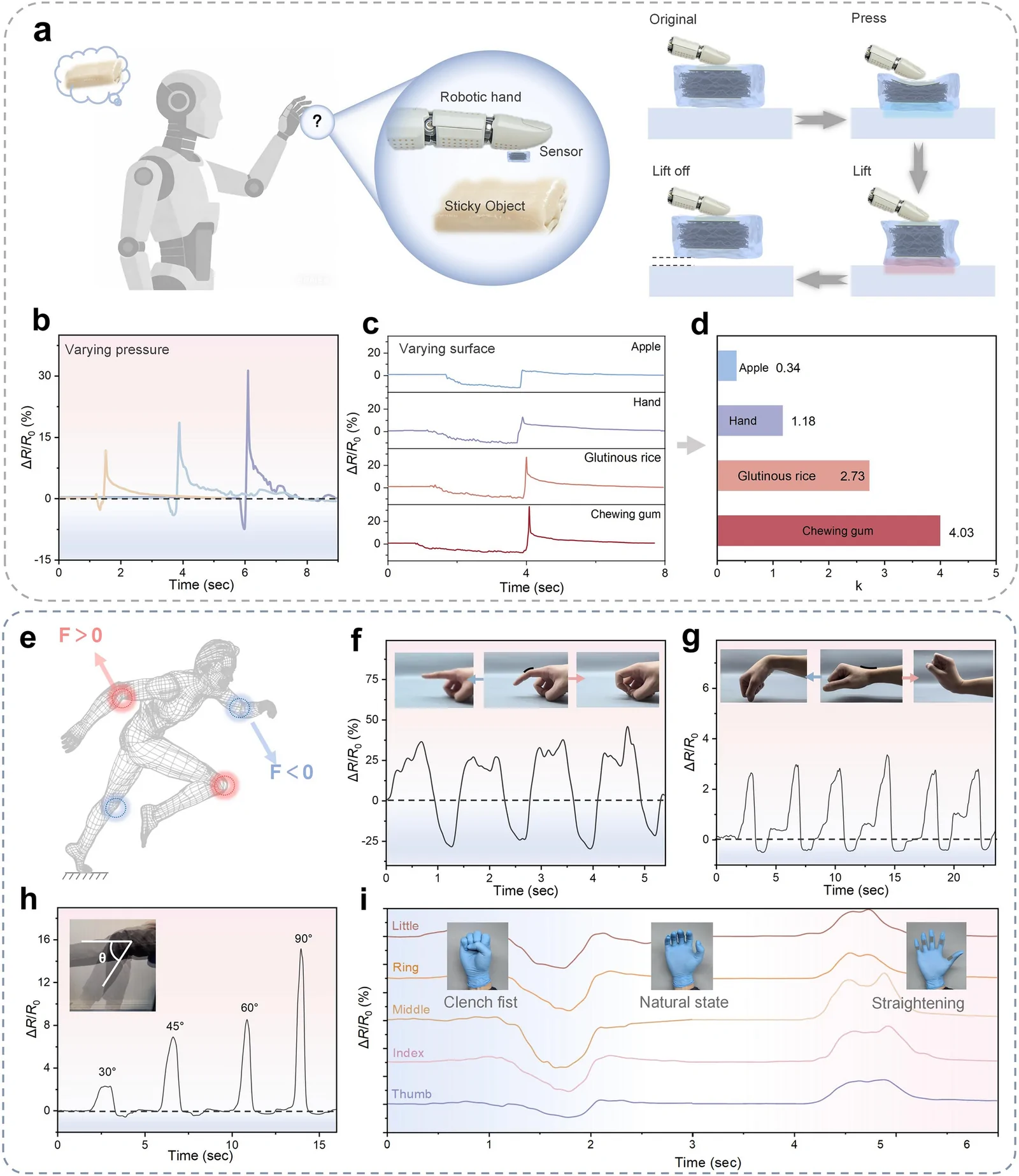
Application demonstration of CNFs-CCFs-A/Dragonskin strain sensors. **a** Sensor response to objects with different surface adhesiveness. **b** Electrical signals generated by the sensor when applying different forces on the same transparent tape surface. **c** Sensor recognition of surfaces with varying stickiness: apple, hand, glutinous rice and chewing gum surfaces, along with **d** the corresponding adhesion-related coefficient *k*. **e** Sensor response to complex movements. **f** Electrical signals during finger bending. **g** Electrical signals during wrist bending and straightening. **h** Motion signals obtained from knee bending and straightening at different angles. **i** Electrical signals during hand flexion and extension, with e-skin modules attached to each finger joint

Due to its stable and robust electromechanical response along a single axial direction, the CNFs-CCFs-A/Dragonskin sensor was employed to monitor motions involving both compression and tension, enabling the recognition of biological surfaces with different adhesion properties. When the sensor was integrated onto a finger and pressed against a sticky surface then followed by lifting, it accurately recorded the compressive response during contact and the tensile response generated during separation between the object and the sensor (Fig. 5a). By repeatedly applying different pressing forces on the same adhesive surface, the interfacial adhesion could be calibrated using the resistance changes during compression and tension. This behavior can be described by the relationship Δ*R*_tension_ = *k*·Δ*R*_compression_, where *k* is a constant (Figs. 5b and S42). The k values for each adhesive tape were obtained by repeated measurements on the same tape surface, and the k values for different tapes were consistent with those measured by conventional mechanical adhesion tests (Fig. S43). These results indicate that the interfacial adhesion force remains constant within the tested range and is independent of the applied pressure, but instead depends on the physical interaction between the adhesive surface and the silicone rubber. Furthermore, the CNFs-CCFs-A/Dragonskin sensor was able to distinguish substrates with different interfacial adhesion strengths. Under the same pressing conditions, distinct Δ*R*_tension_ signals were observed during separation from apple peel, human skin, glutinous rice, and chewing gum surfaces (Fig. 5c). Based on the fitted relationship, the corresponding adhesion coefficients *k* were calculated to be 0.34, 1.18, 2.73, and 4.03, respectively (Fig. 5d). These results demonstrate the potential of the sensor for environmental perception in wearable systems.

In addition to adhesion sensing, the CNFs-CCFs-A/Dragonskin sensor also functioned as a deformable conductor for human motion monitoring (Fig. 5e). When attached to the finger (Fig. 5f) and wrist (Fig. 5g), the sensor generated stable and periodic electrical signals that clearly reflected the direction and magnitude of motion. Typical daily movements, such as knee bending and straightening (Fig. 5h), were also well captured through periodic current responses, with the bending angle clearly distinguished by the signal amplitude. To demonstrate practical wearable applications, the CNFs-CCFs-A/Dragonskin sensor was integrated onto an adult’s right hand to enable real-time signal collection under three representative states: a natural state, finger extension and a clenched fist (Fig. 5i). The resulting signals were sensitive, directional, and highly reproducible, highlighting the capability of the sensor for fine motion detection and its promise for future wearable electronic skin and human–machine interaction systems.

## Discussion

In summary, we have developed a hierarchical carbon aerogel (CNFs-CCFs-A) for coaxial bidirectional strain sensing by integrating catalyst-interface engineering with a sustainable carbon-source strategy. By employing biomass-derived cotton fibers as an elastic scaffold, Ni-S_*x*_ as a deactivation-resistant catalyst, and polyolefin plastics as a sustained-release carbon source, uniform and controllable growth of carbon nanofibers is achieved on carbonized cotton fibers. This approach effectively suppresses catalyst encapsulation and uneven carbon deposition, enabling the construction of a structurally consistent multilevel conductive network.

The resulting CNFs-CCFs-A exhibits excellent elastic resilience and stable electromechanical performance under both tensile and compressive deformations. Owing to the synergistic modulation of contact conductance and tunneling conductance within the hierarchical network, the sensor demonstrates a wide bidirectional working window, high sensitivity, and good linearity, together with long-term durability over more than 2000 tension–compression cycles. More importantly, the single-layer sensing architecture allows reliable discrimination of strain direction and magnitude without the need for complex multilayer designs or signal decoupling strategies.

Beyond fundamental strain sensing, the CNFs-CCFs-A/Dragonskin device shows versatile application potential, including precise monitoring of human joint motions and recognition of biological surface adhesion. These capabilities highlight the advantages of hierarchical carbon architectures in multifunctional wearable sensing. Overall, this work provides a scalable and sustainable strategy for designing reconfigurable carbon networks and offers new insights into the development of high-performance bidirectional strain sensors for next-generation wearable electronics and human–machine interfaces.

## Supplementary Information

Below is the link to the electronic supplementary material.

Supplementary file1 (DOCX 20248 kb)
