# Supplementary material for: Sustainable Carbon Aerogels from Polyolefin Plastics for High-Linearity Bidirectional Strain Sensing
Source: Nanomicro Lett. 2026 May 9;18:362. doi: 10.1007/s40820-026-02196-7 (PMC13156365; doi:10.1007/s40820-026-02196-7)
Supplement: Supplementary file 1 — Supplementary file1 (DOCX 20248 kb) [file 40820_2026_2196_MOESM1_ESM.docx]

Supporting Information for

**Sustainable Carbon Aerogels from Polyolefin Plastics for High-Linearity Bidirectional Strain Sensing**

Yang Yue^1, 2^, Hui Bi^1, 4,^ *, Shiyu Zhang^1, 2^, Chen Luan^3^, Zhangliu Tian^1, 2^, Dayong Ren^1, 2^, Fuqiang Huang^4,^ *

^1^ State Key Laboratory of High-Performance Ceramics, Shanghai Institute of Ceramics, Chinese Academy of Sciences, Shanghai 200050, P. R. China

^2^ Center of Materials Science and Optoelectronics Engineering, University of Chinese Academy of Sciences, Beijing 100049, P. R. China

^3^ Zhejiang Key Laboratory of Industrial Solid Waste Thermal Hydrolysis Technology and Intelligent Equipment, Huzhou University, Huzhou 313000, P. R. China

^4^ Key Laboratory of Intelligent Creation for Extreme Energy Materials of Ministry of Education, School of Materials Science and Engineering and Zhangjiang Institute for Advanced Study, Shanghai Jiao Tong University, Shanghai 200240, P. R. China

* Corresponding authors. E-mail: [huangfq@sjtu.edu.cn](mailto:huangfq@sjtu.edu.cn) (Fuqiang Huang); [bihui@sjtu.edu.cn](mailto:bihui@sjtu.edu.cn) (Hui Bi)

**Supplementary Figures**


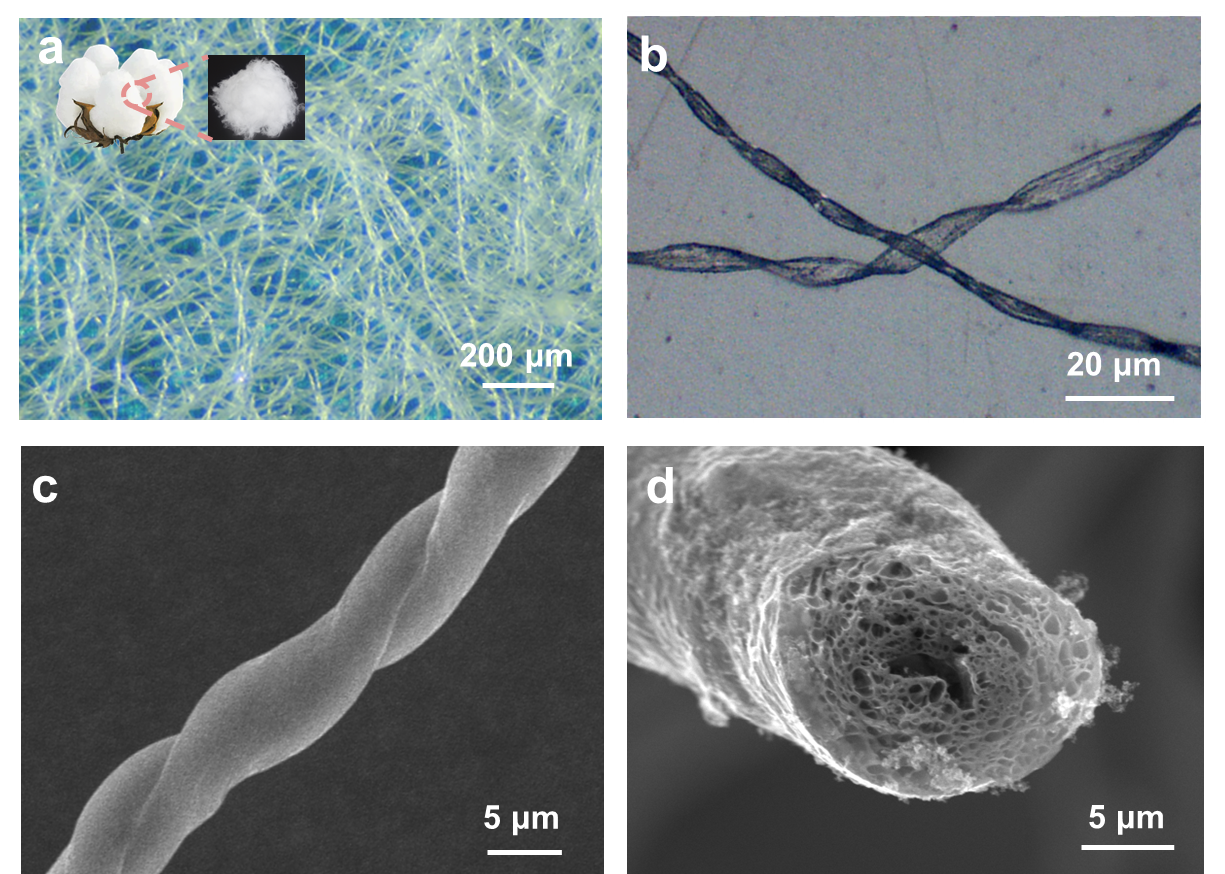


**Fig. S1 (a)** Optical microscopy image of cotton fibers. **(b)** Magnified optical microscopy image of cotton fibers after carbonization at 850 °C, together with the corresponding **(c)** scanning electron microscopy (SEM) image. **(d)** high-resolution SEM image of the cross section of the cotton fibers.


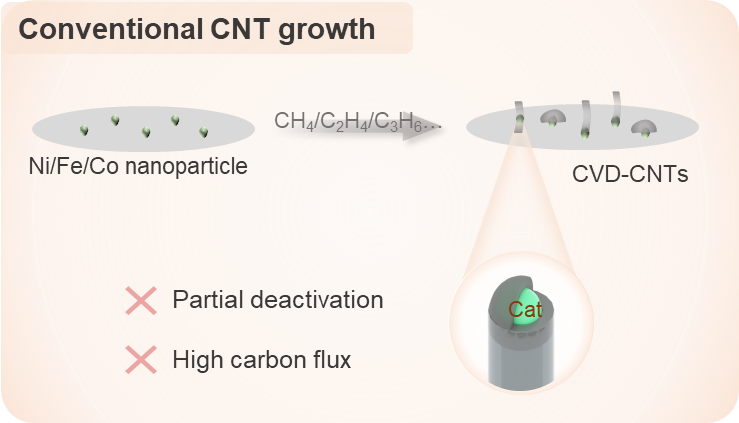


**Fig. S2** Schematic illustration of the catalyst deactivation mechanism during CNFs synthesis using conventional Ni/Fe/Co catalysts


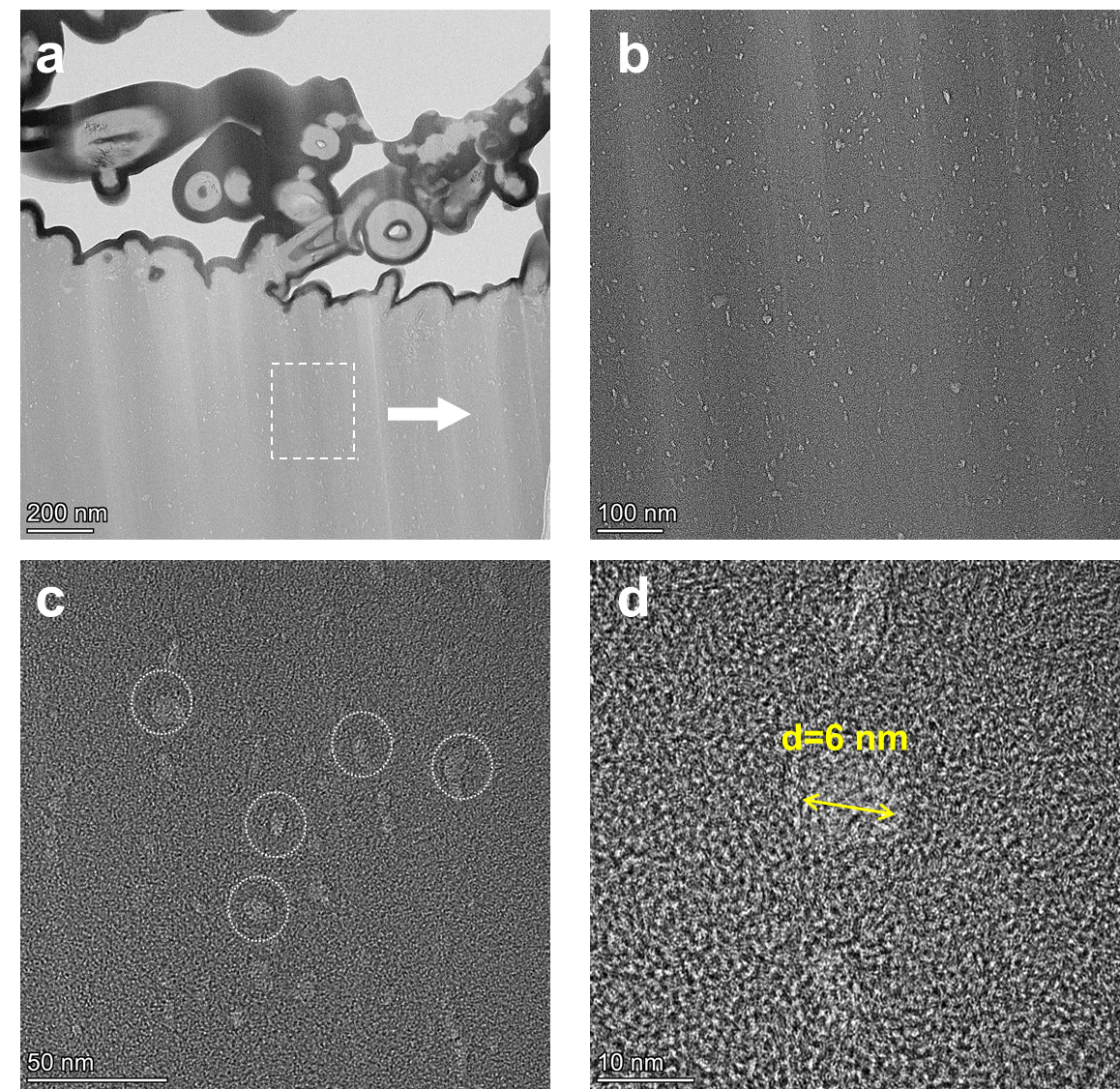


**Fig. S3 (a)** Low-magnification TEM image of the CNFs-CCF cross section prepared by focused ion beam (FIB). **(b-d)** Enlarged TEM images of the selected region

**Fig. S4** X-ray diffraction (XRD) pattern of carbonized cotton fibers (CCF) obtained at 850 °C


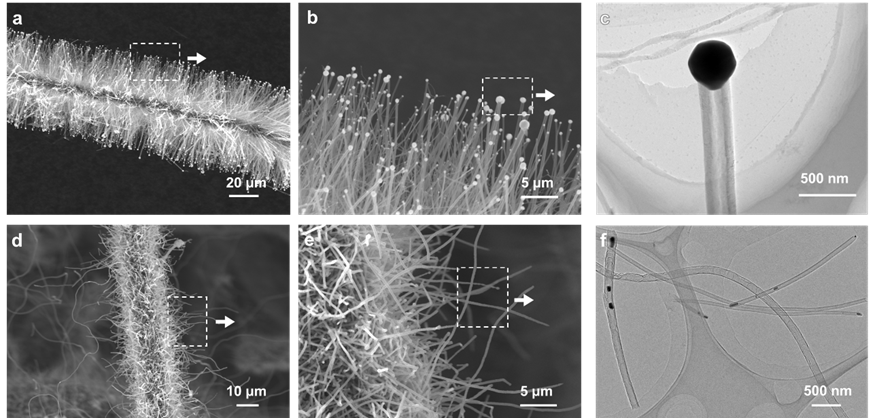


**Fig. S5 (a, b)** High-resolution SEM images and **(c)** TEM image of Ni-S_x_-CNFs-CCF. **(d, e)** High-resolution SEM images and **(f)** TEM image of Ni-CNFs–CCF


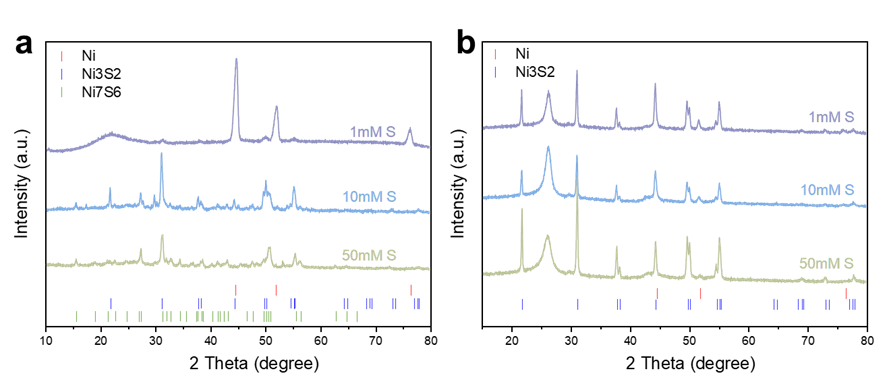


**Fig. S6 (a)** XRD patterns of cotton loaded with 10 mM Ni(NO_3_)_2_ under different sulfur fluxes before introducing the carbon source, and **(b)** the corresponding XRD patterns after 2 h of reaction with the carbon source


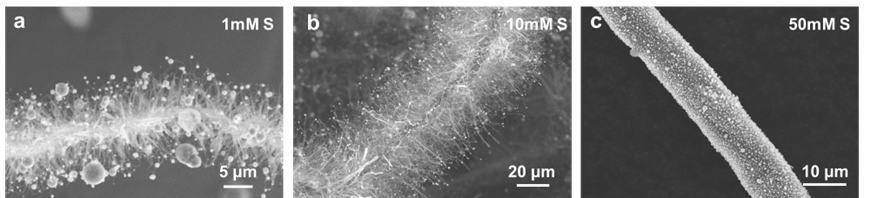


**Fig. S7** SEM images of cotton loaded with 10 mM Ni(NO_3_)_2_ after 2 h of reaction with the carbon source under different sulfur fluxes: **(a)** 1 mM, **(b)** 10 mM, and **(c)** 50 mM


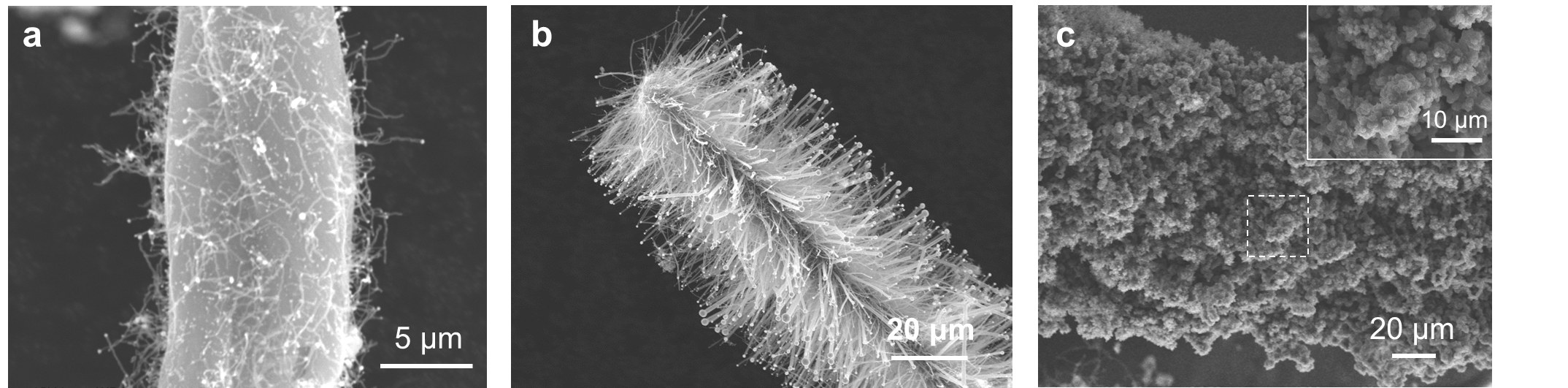


**Fig. S8** SEM images of Ni-S_x_-CNF-CCF prepared at **(a)** 700 °C, **(b)** 850 °C, and **(c)** 1000 °C

**Fig. S9** XRD patterns of Ni-S_x_-CNFs-CCF prepared at 700 °C, 850 °C, and 1000 °C

**
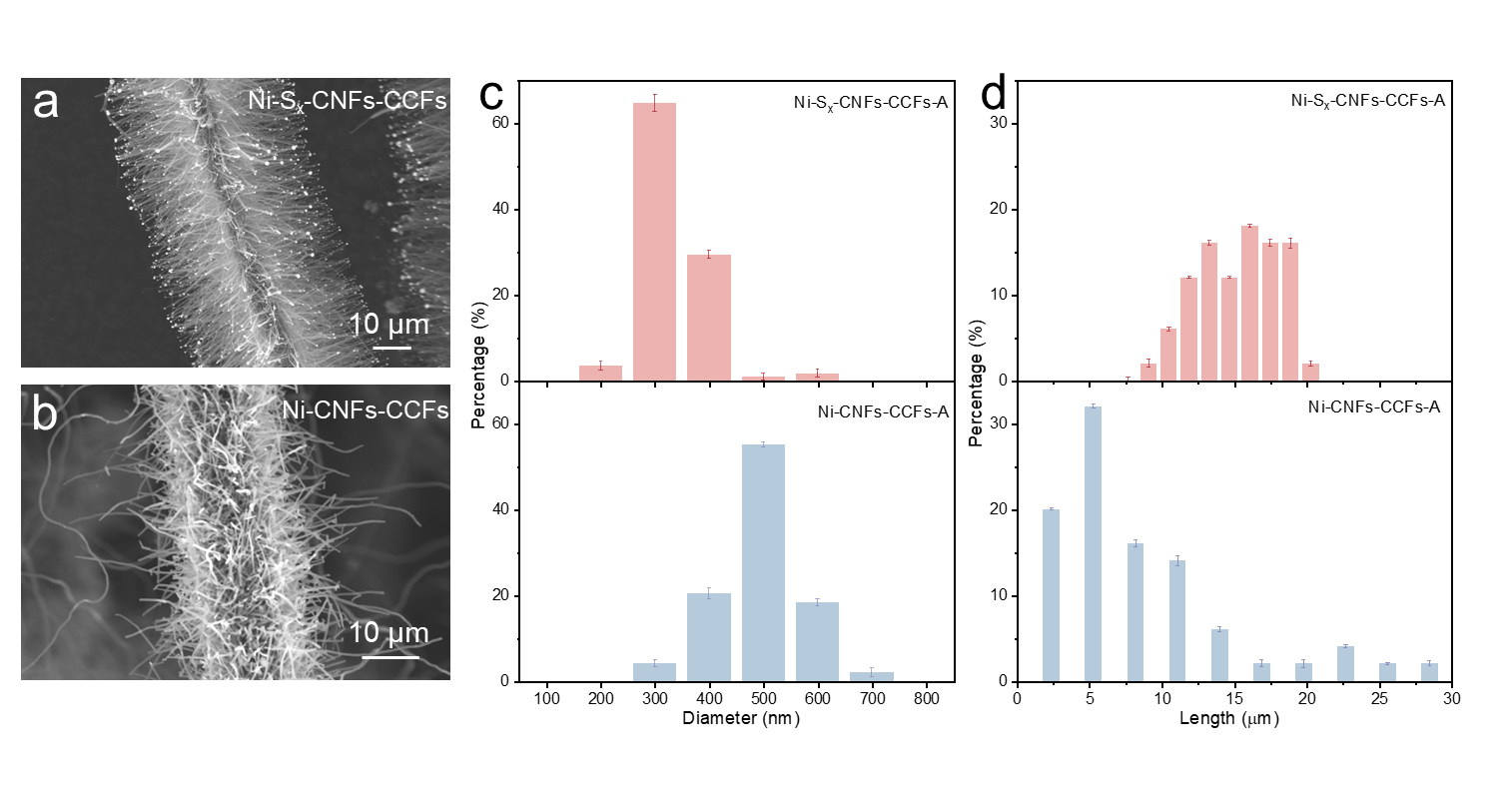
**

**Fig. S10 (a)** SEM image of Ni-S_x_-CNFs-CCFs and **(b)** SEM image of Ni-CNFs-CCFs, along with the corresponding **(c)** CNFs diameter distribution and **(d)** CNFs length distribution statistics (with error bars)


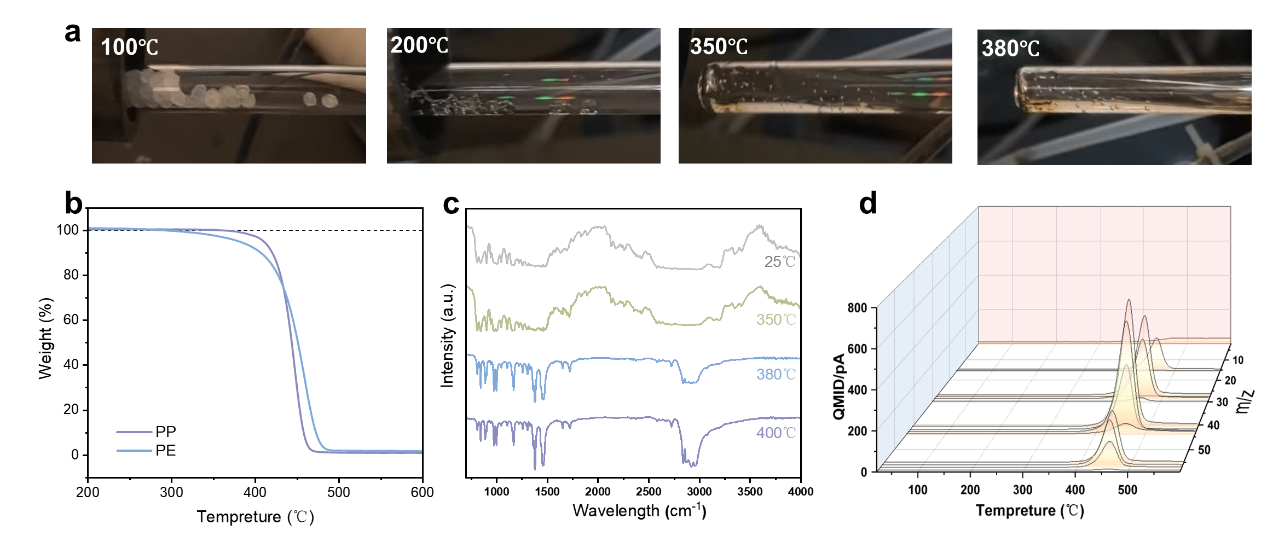


**Fig. S11** (**a**) Optical photographs showing the evolution of a PP/PE (1:1) mixture during heating. (**b**) Thermogravimetric (TG) curves, (**c**) FTIR spectra, and (**d**) mass spectra of gaseous small molecules generated during pyrolysis


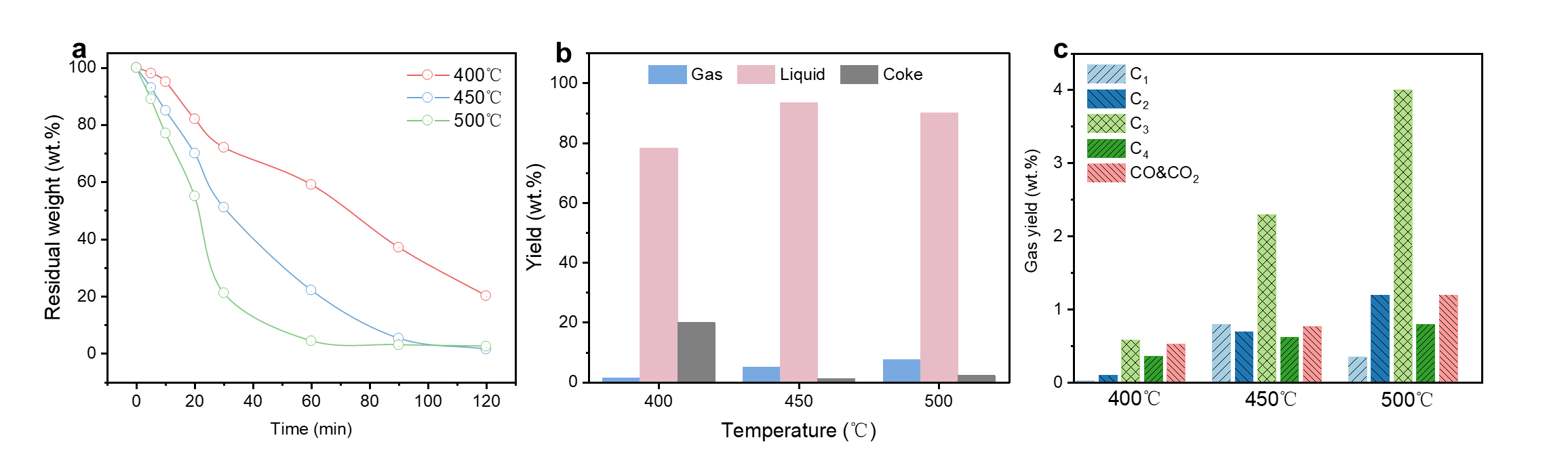


**Fig. S12 (a)** Residual solid mass of PP/PE as a function of time at different temperatures. **(b)** Yields of gas, liquid, and solid products at different temperatures, and **(c)** corresponding gas product yields


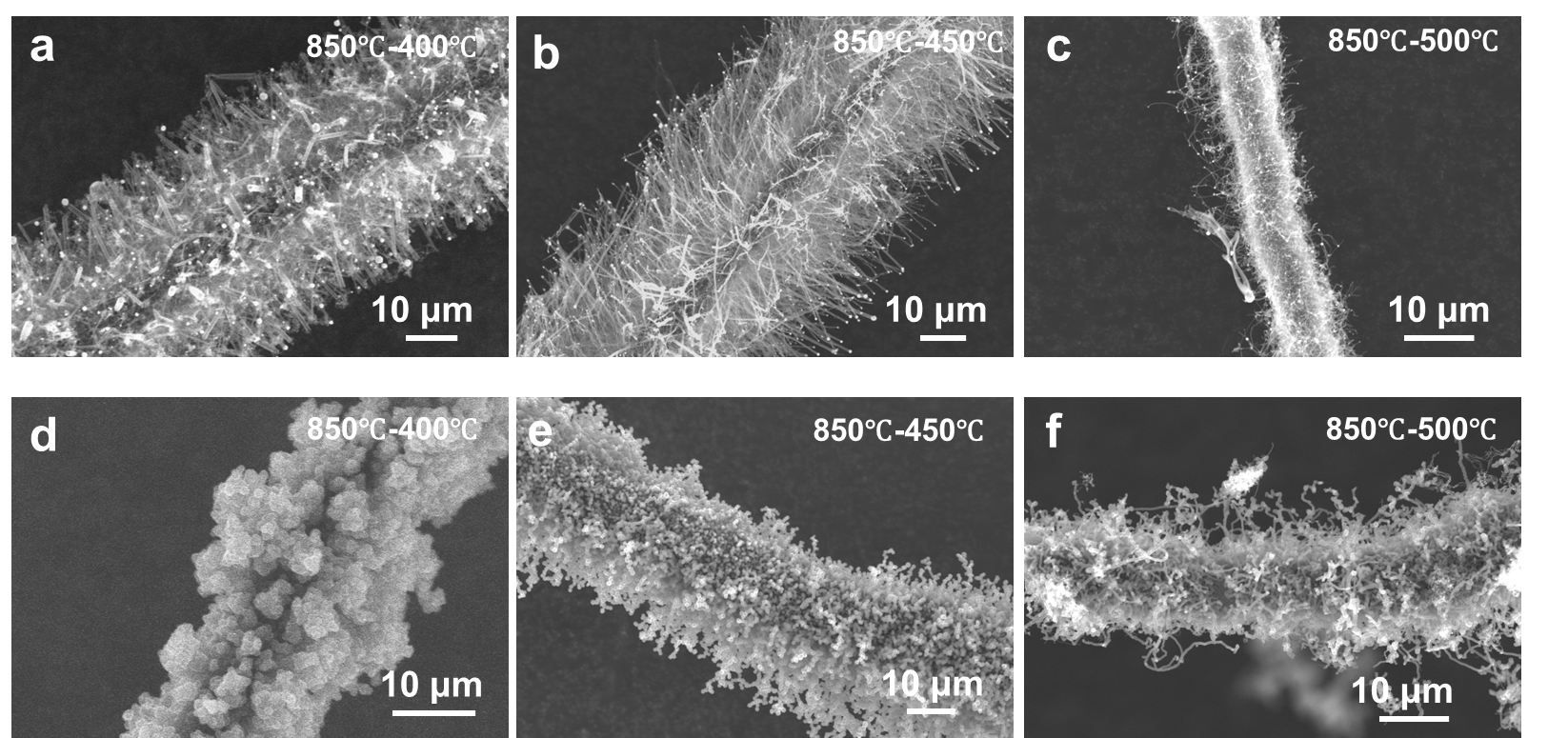


**Fig. S13** Ni-S_x_-CNFs-CCF prepared using PP/PE at **(a)** 400 °C, **(b)** 450 °C, and **(c)** 500 °C. Ni-CNFs-CCF prepared using PP/PE at **(d)** 400 °C, **(e)** 450 °C, and **(f)** 500 °C


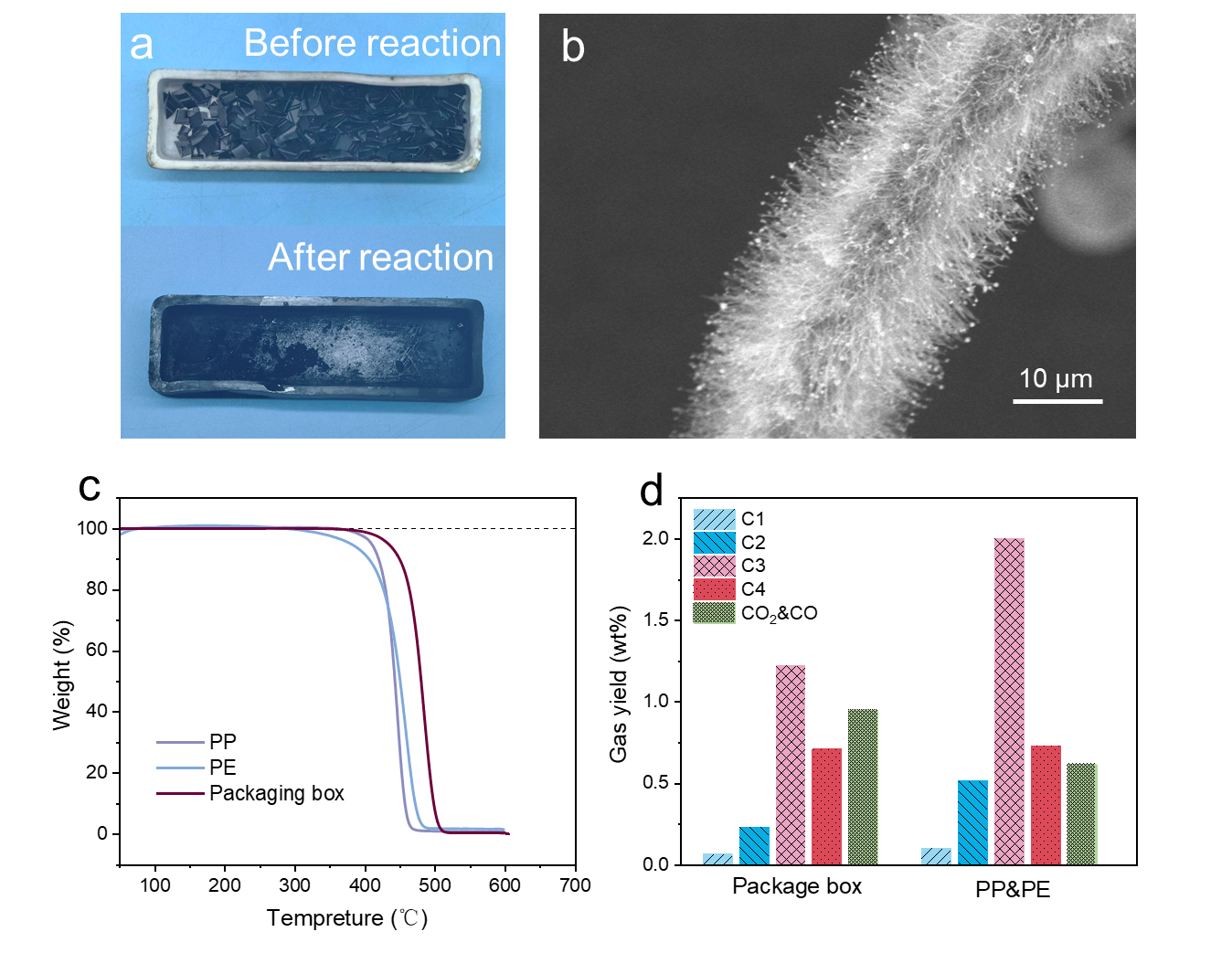


**Fig. S14 (a)** Optical photographs of the CNFs catalytic growth reaction using a post-consumer black PP food packaging container as the carbon source, taken before and after the reaction. **(b)** SEM images of the resulting CNFs-CCFs. **(c)** TGA curves of post-consumer waste plastic packaging boxes, and **(d)** gas chromatography (GC) analysis of the gaseous pyrolysis products at 500 °C


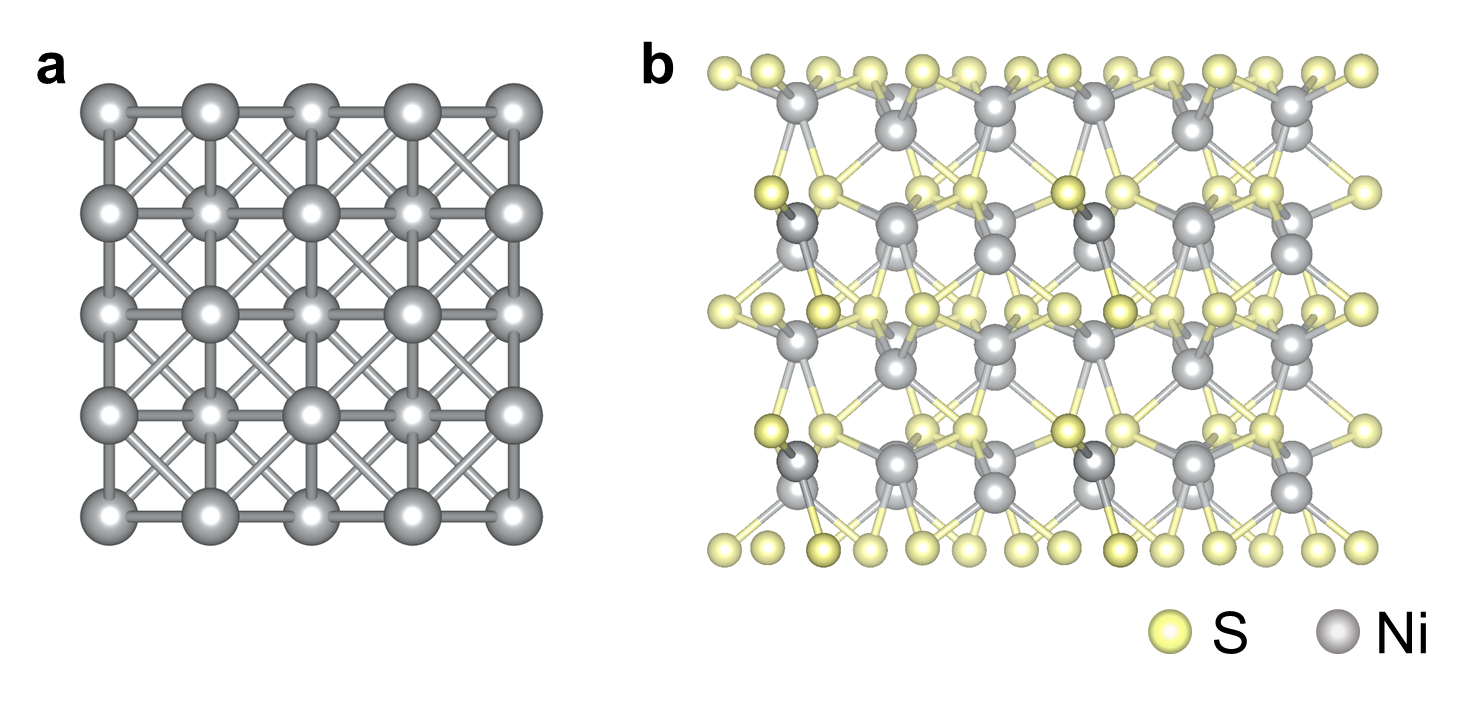


**Fig. S15** Crystal structures of **(a)** Ni and **(b)** Ni_3_S_2_

**
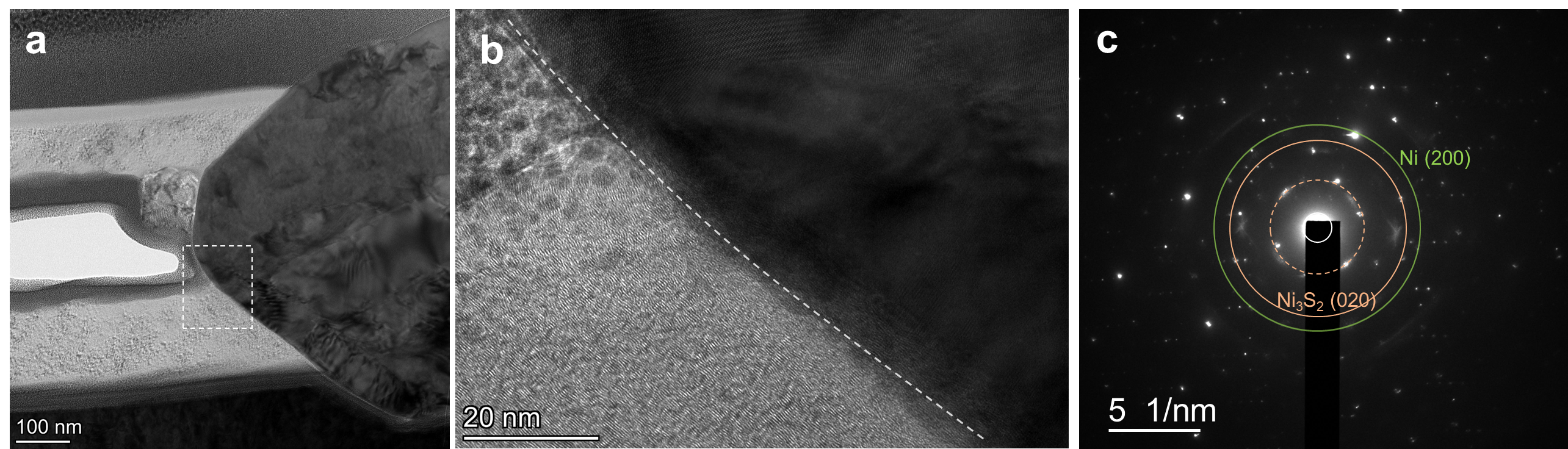
**

**Fig. S16 (a)** TEM image of Ni-S_x_-CNF, **(b)** enlarged TEM image of the corresponding region, and **(c)** the corresponding selected area electron diffraction (SAED) pattern


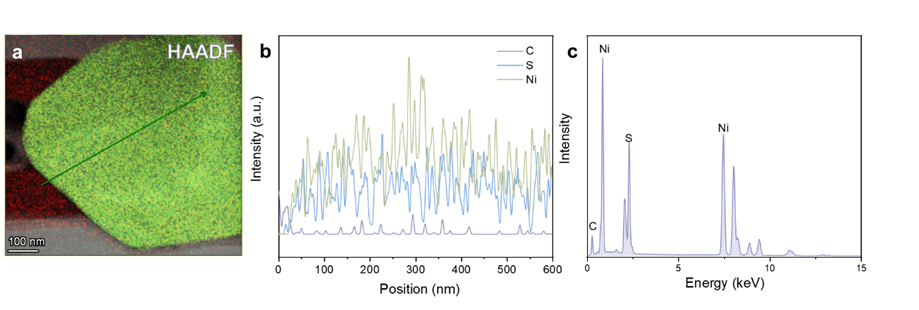


**Fig. S17 (a)** HAADF-STEM image of Ni-S_x_-CNF after FIB processing, and **(b)** corresponding elemental line-scan profiles along the green line. **(c)** Energy-dispersive spectroscopy (EDS) of the region


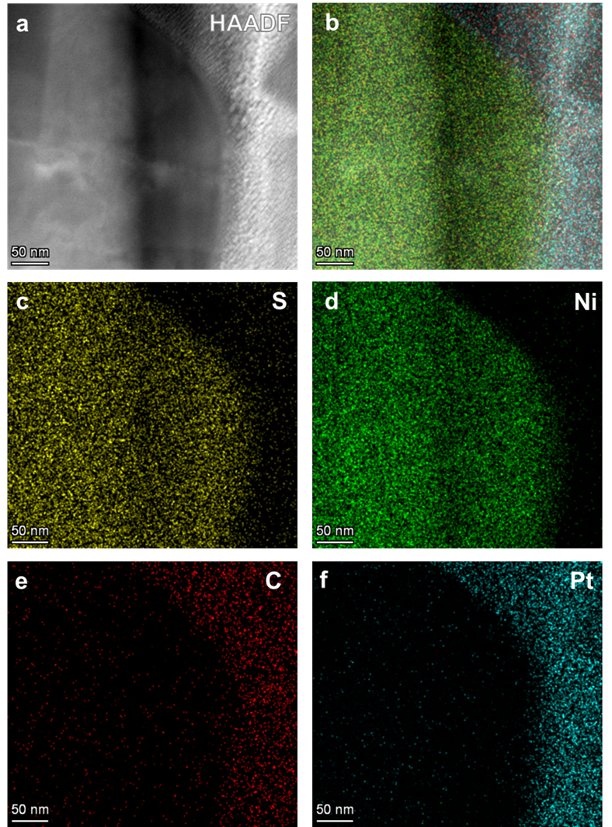


**Fig. S18** HAADF-STEM images of Ni-S_x_-CNF after FIB processing and the corresponding elemental mappings

**Fig. S19** Raman spectrum of CCF carbonized at 850 °C


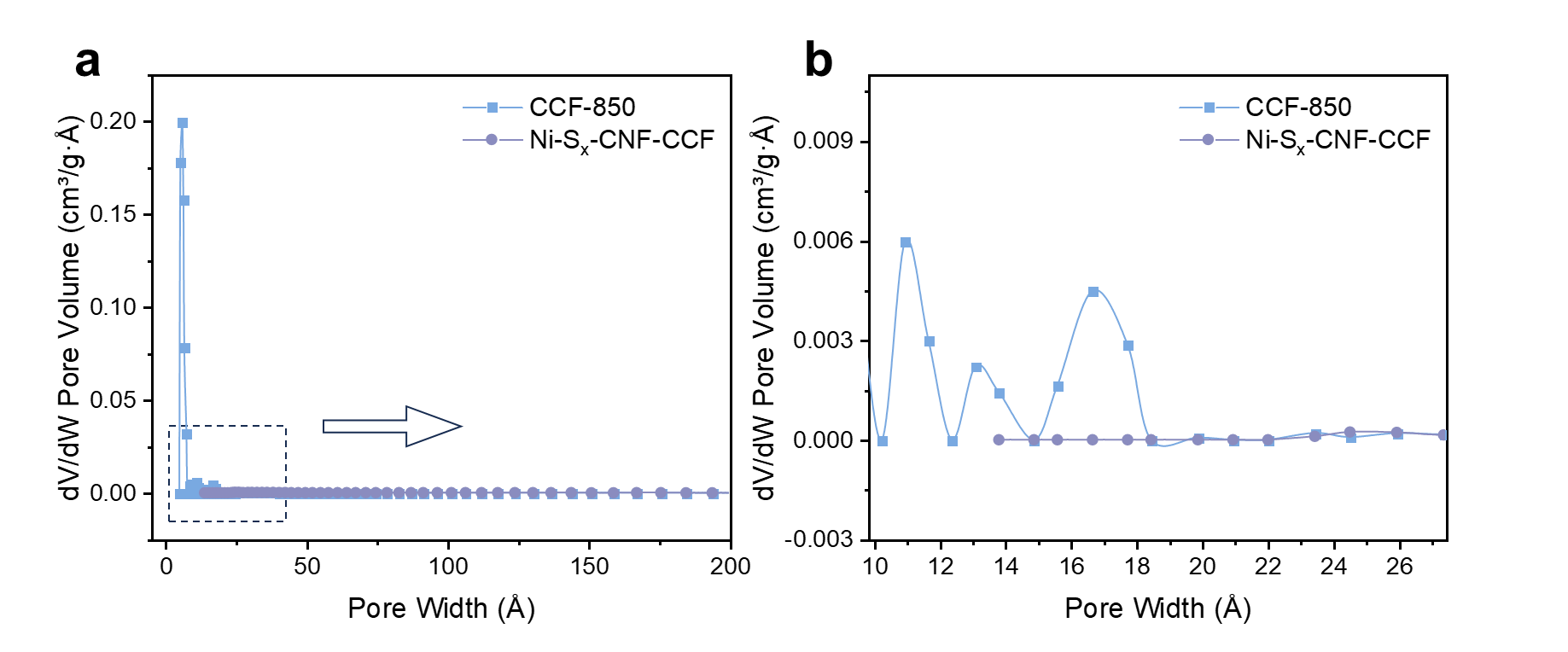


**Fig. S20 (a)** Pore size distributions and **(b)** enlarged views of Ni-S_x_-CNF-CCF and CCF carbonized at 850 °C


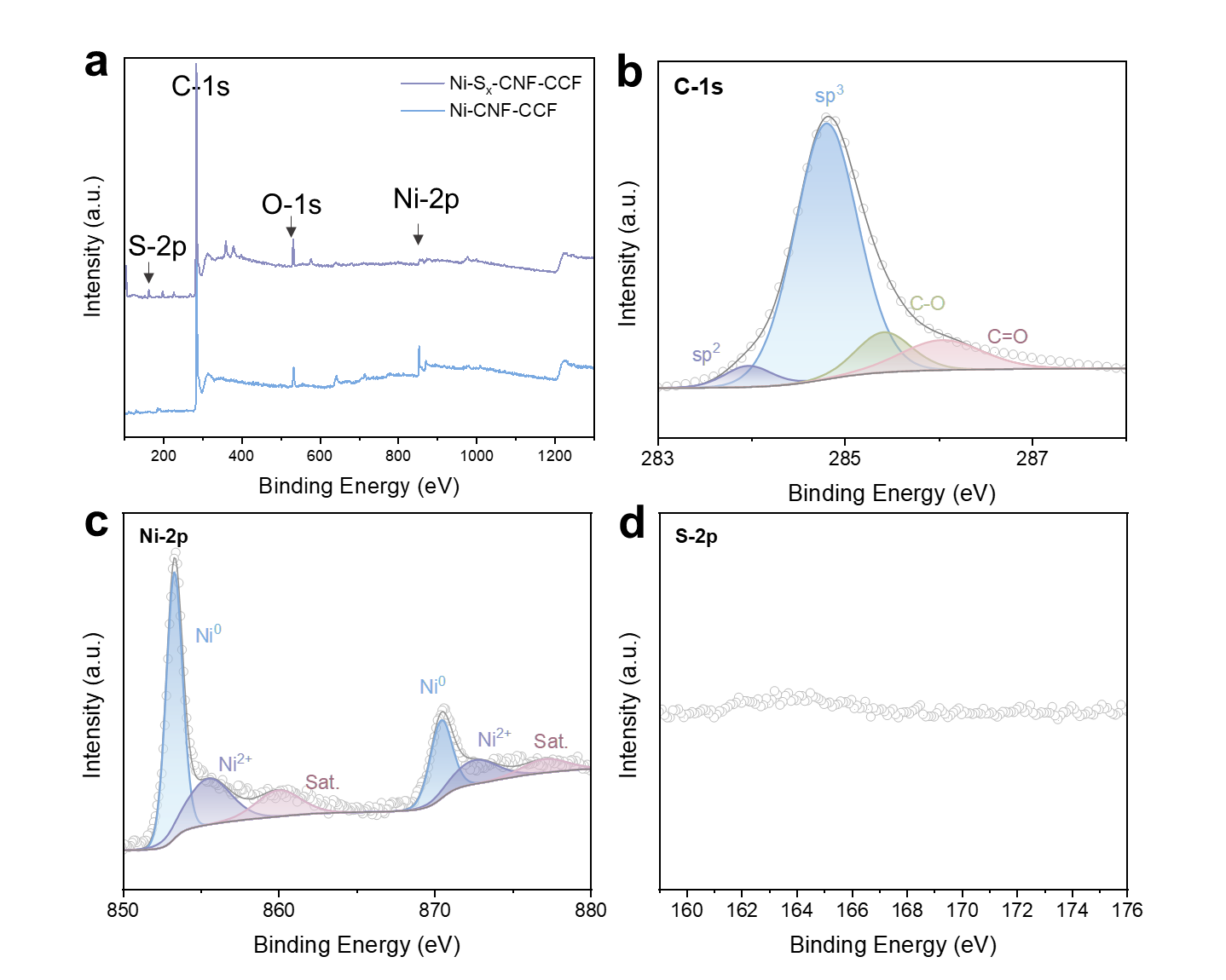


**Fig. S21 (a)** XPS survey spectra of Ni-S_x_-CNF-CCF and Ni-CNF-CCF. **(b-d)** High-resolution C 1s, Ni 2p, and S 2p spectra of Ni-CNF-CCF

**Fig. S22** FTIR spectra of Ni-S_x_-CNF-CCF and Ni-CNF-CCF


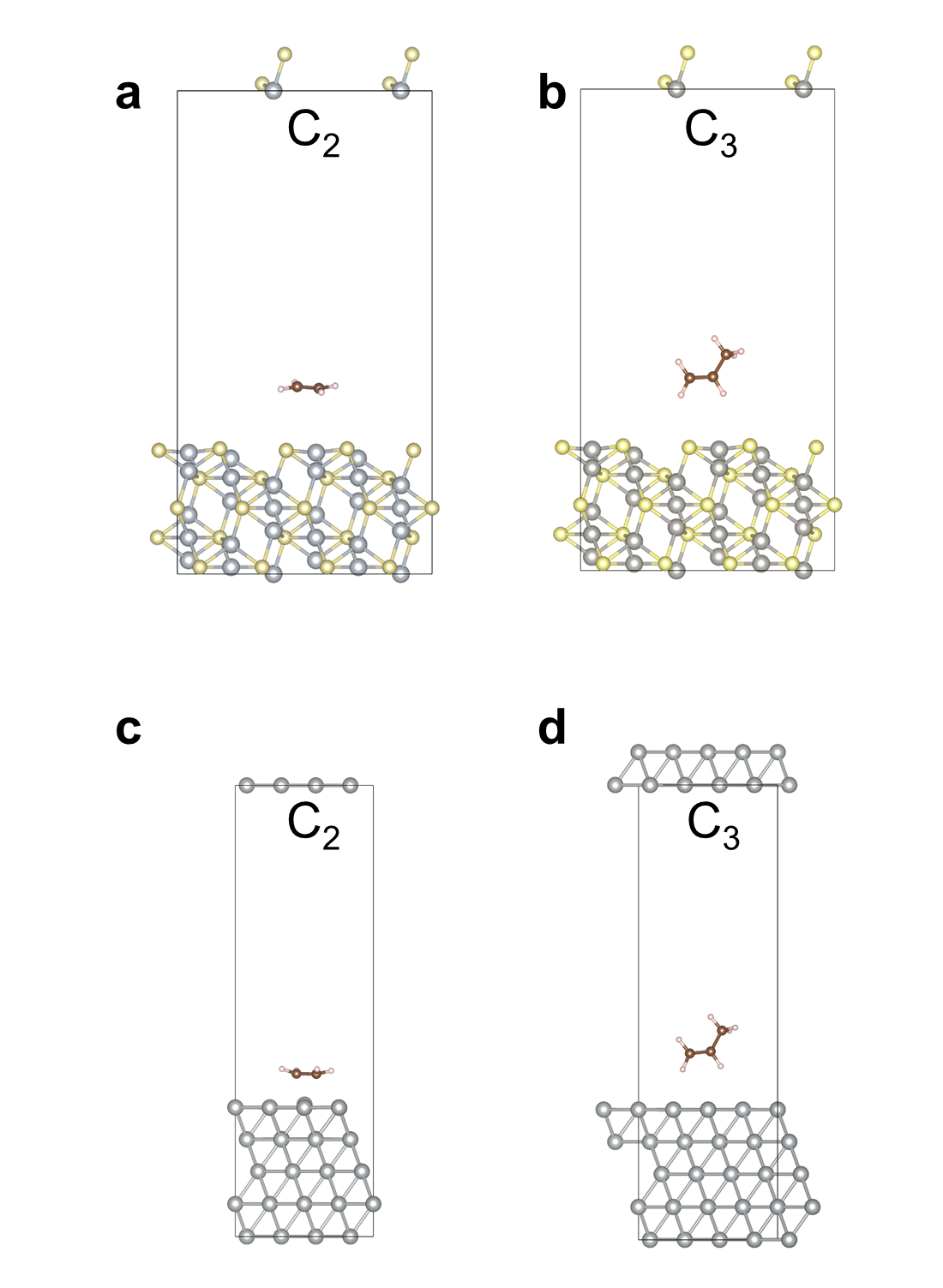


**Fig. S23** DFT models of **(a)** C_2_ and **(b)** C_3_ adsorption on the Ni_3_S_2_ (020) surface, and **(c)** C_2_ and **(d)** C_3_ adsorption on the Ni (111) surface


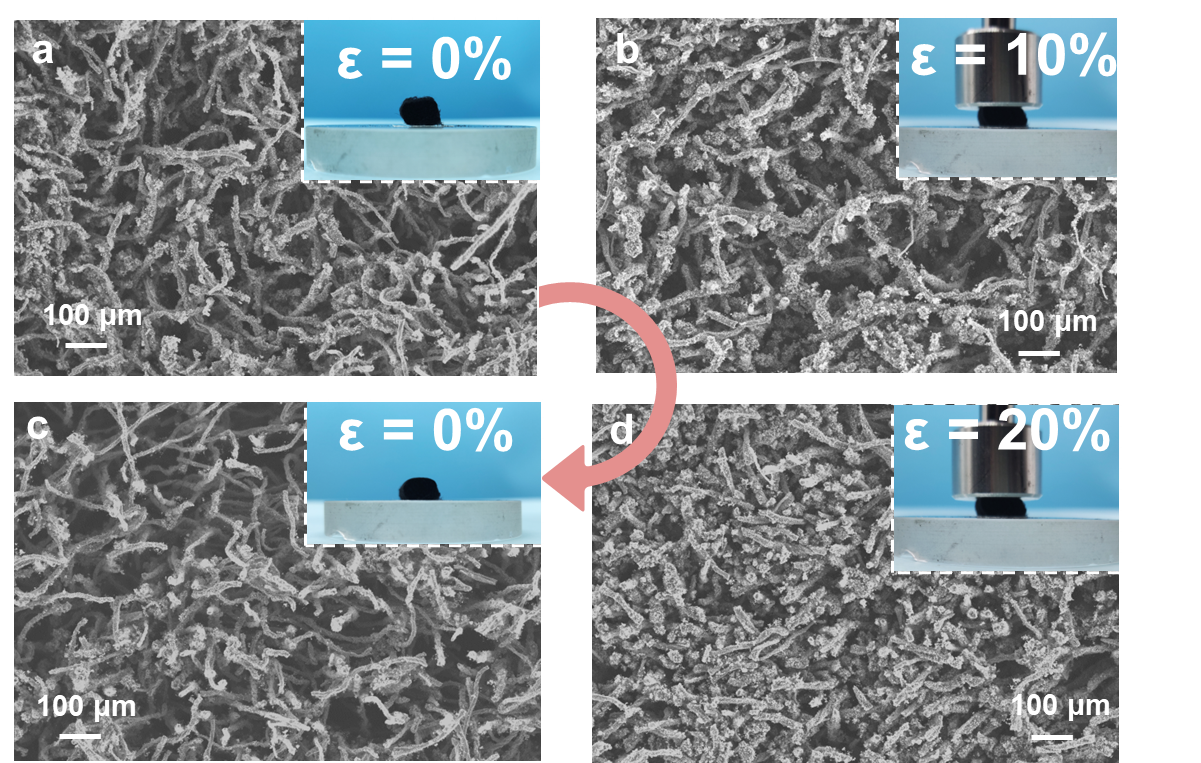


**Fig. S24** SEM images of CNFs-CCFs-A restored after 20% compression. Insets are the corresponding macroscopic optical images


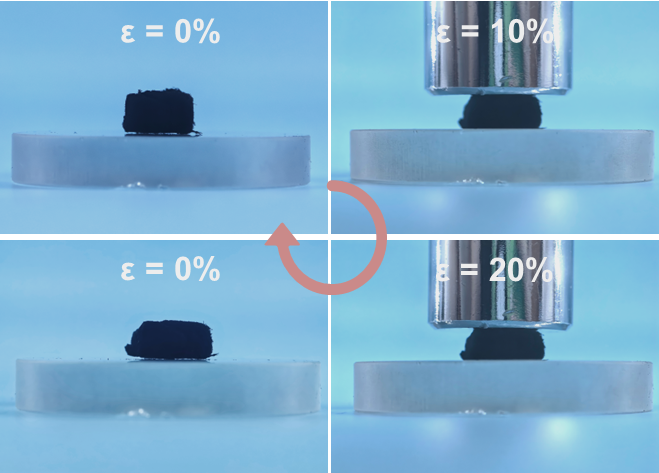


**Fig. S25** Optical photographs of CNFs-CCFs-A with 60mg·cm^-3^ density after unloading stress following 20% strain

**Fig. S26** Bulk electrical conductivity of CNFs-CCFs-A at densities of 13, 20, 35, and 60 mg cm^-3^

**Fig. S27** Stress-strain curves of CNFs-CCFs-A with a density of 20 mg cm^-3^ during cyclic compression at 40% strain


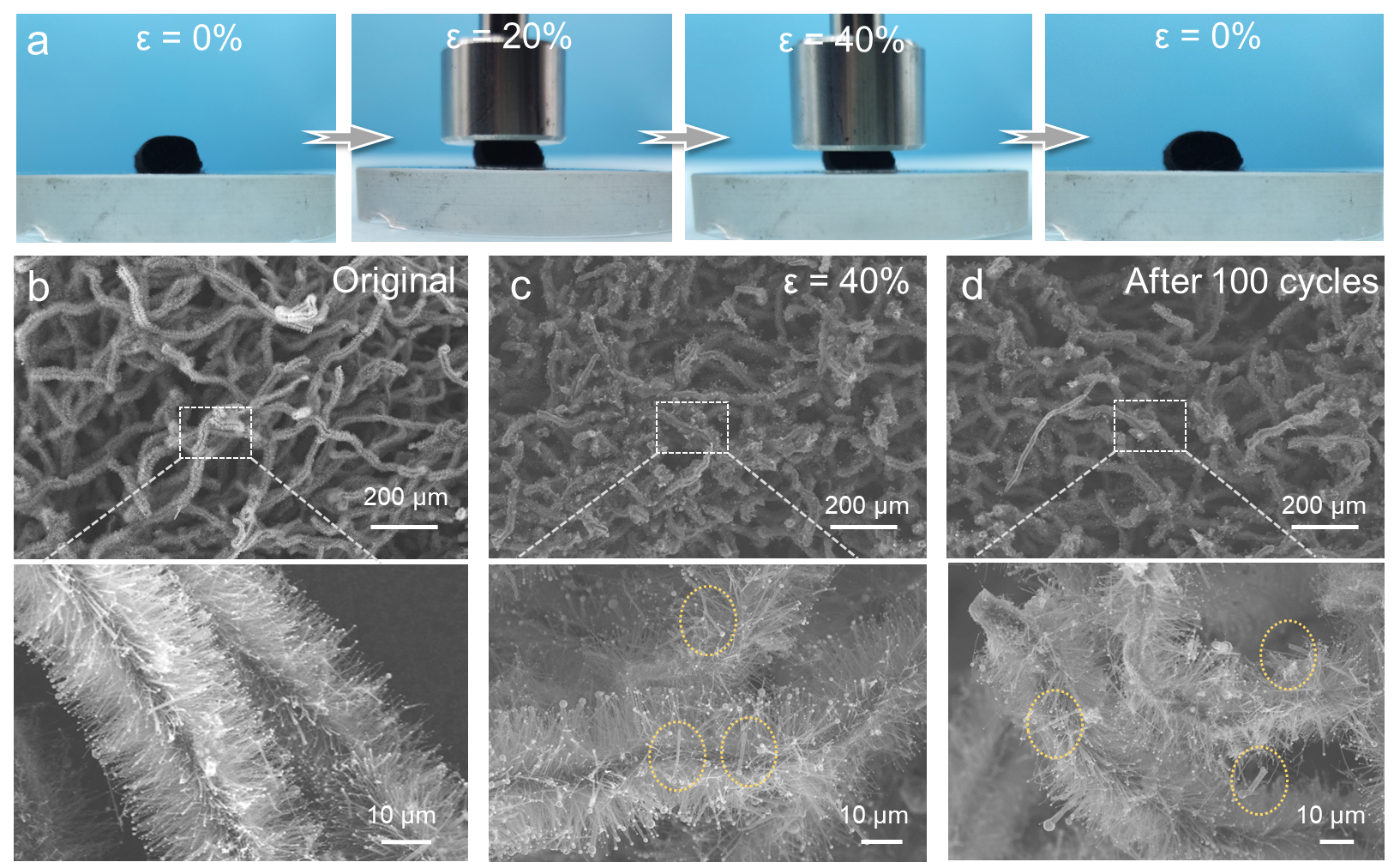


**Fig. S28 (a)** Optical photographs of the aerogel during a compression–release process. (b-d) SEM images of the CNFs-CCFs: **(b)** original, **(c)** compressed to 40% strain, and **(d)** after 100 compression cycles at 40% strain. The lower panels show magnified views of the boxed regions, yellow dashed circles mark localized structural changes after high-strain compression


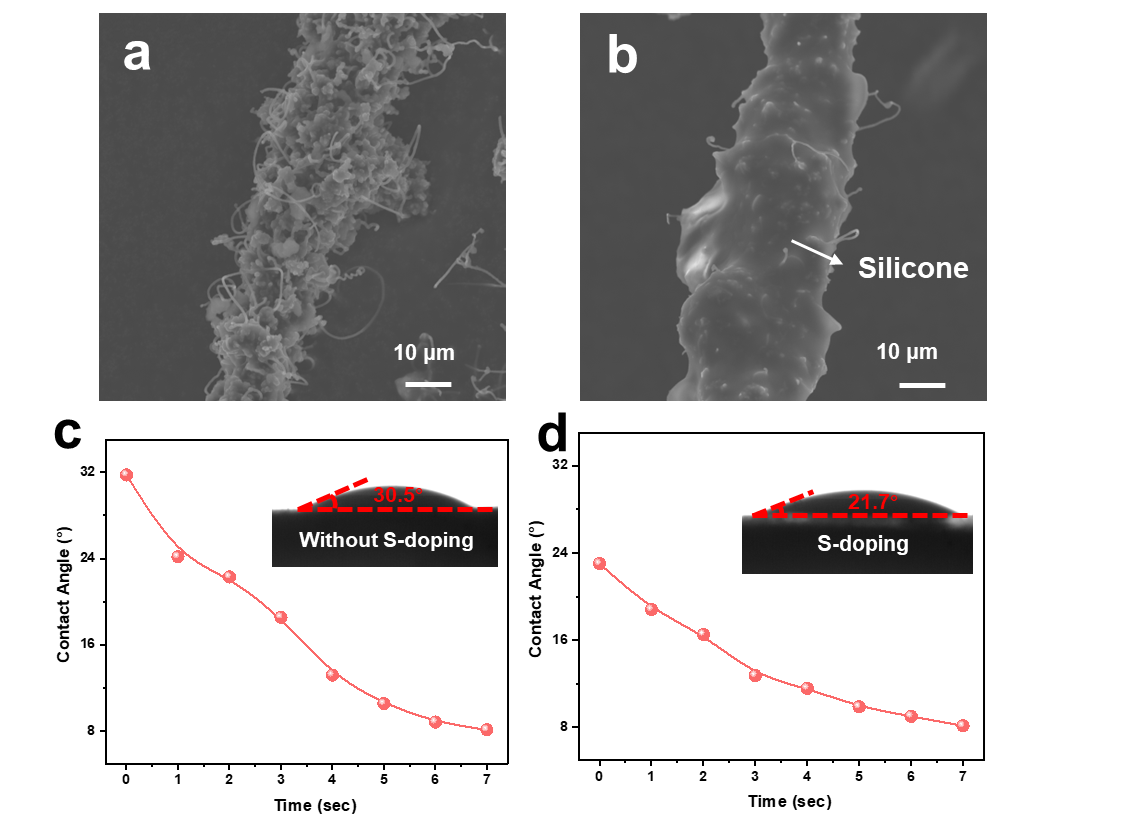


**Fig. S29** Comparison of SEM images of **(a)** without S-doping CNFs-CCF and **(b)** S-doping CNFs-CCF after soaking in 15 wt.% Dragonskin in n-hexane solution. Dynamic contact angles of **(c)** without S-doping CNFs-CCF and **(d)** S-doping CNFs-CCF with 15 wt.% Dragonskin in n-hexane solution


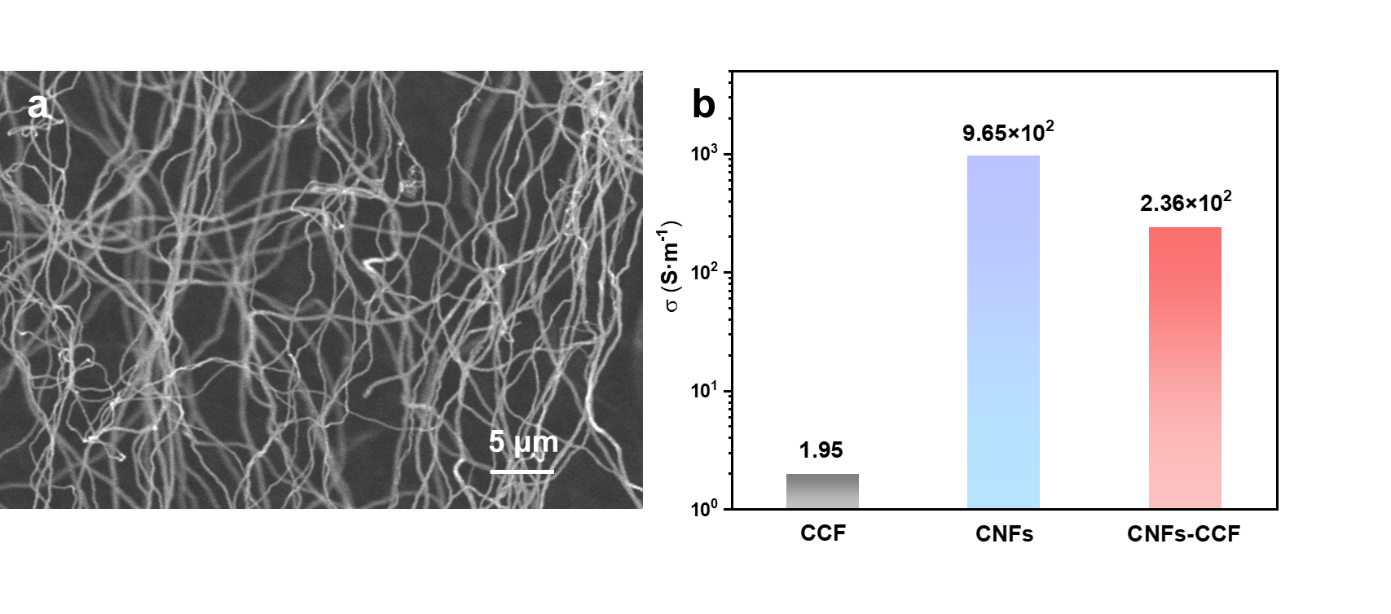


**Fig. S30 (a)** SEM image of carbon nanofibers (CNFs) catalytically grown by loading Ni-S_x_ on Al_2_O_3_ crucible with CCFs-CNFs under the same atmosphere and temperature condition. **(b)** Comparison of conductivity of each component


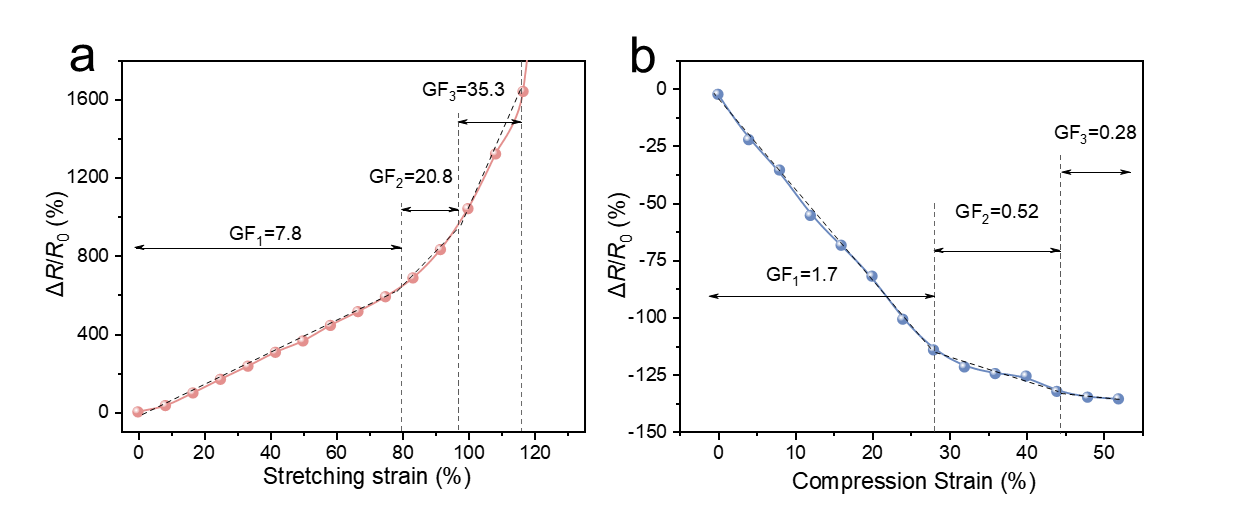


**Fig. S31** (**a**) Stretch-recovery response and GF of the composite under tensile strains from 0% to 100% (**b**) compression-recovery response and GF of the composite under compressive strains from 0 to 40%


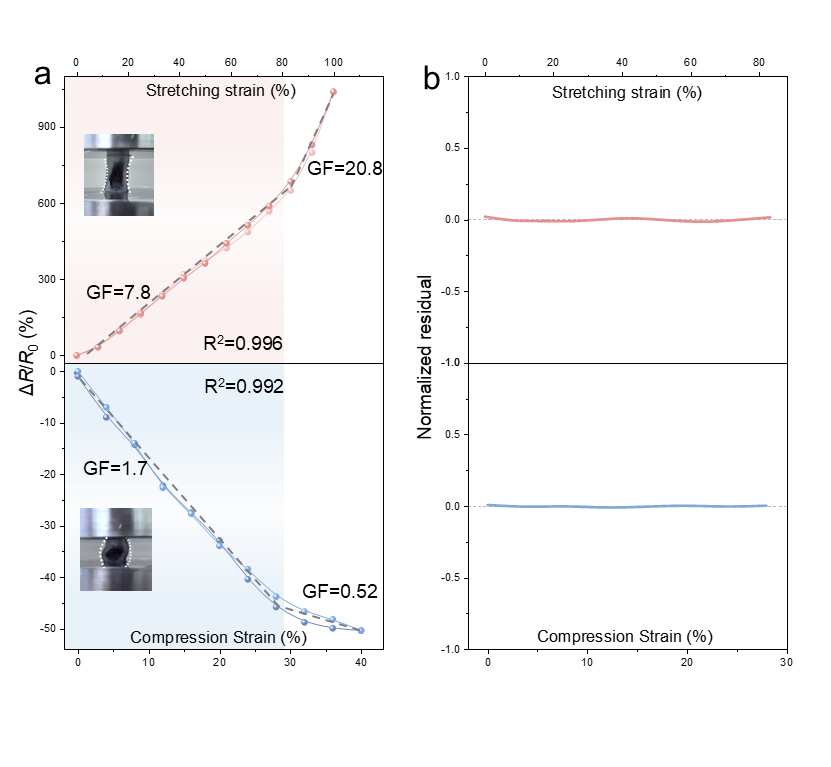


**Fig. S32 (a)**Stretch-recovery response of the composite under tensile strains from 0% to 100% and compression-recovery response of the composite under compressive strains from 0% to 40%, the colored regions indicate the defined linear ranges. **(b)** Corresponding normalized residual plots


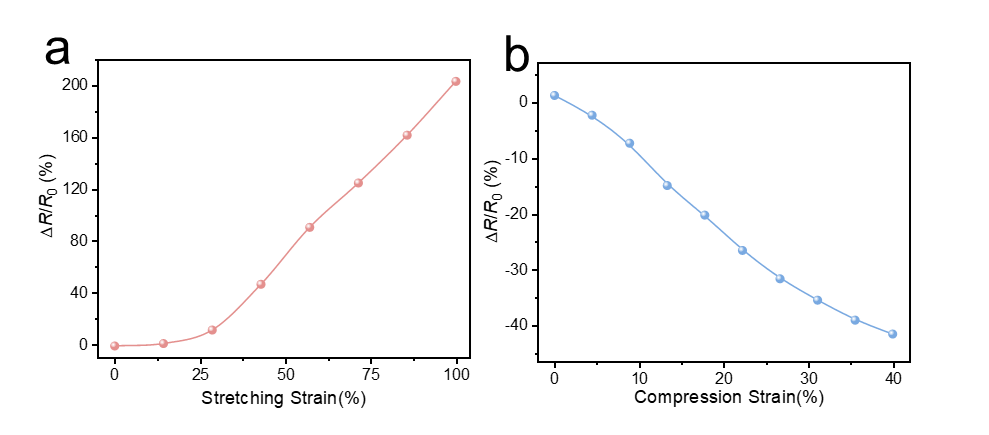


**Fig. S33** Relative resistance change of CCF-A under (**a**) tensile strain and (**b**) compressive strain.


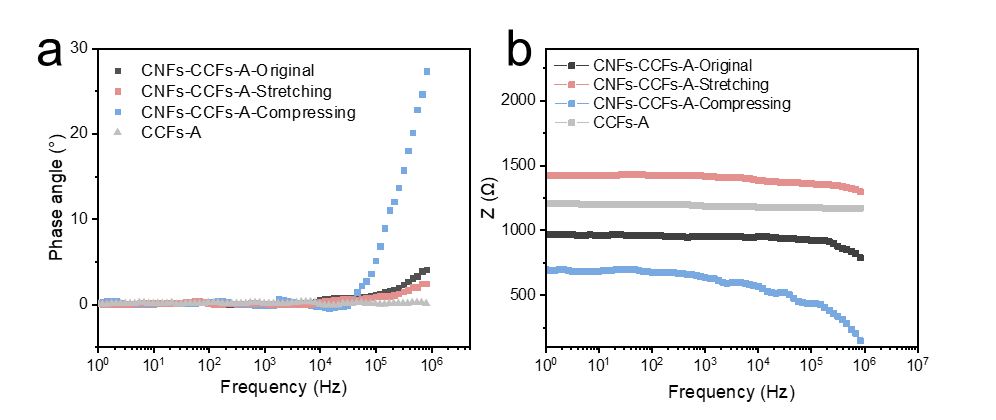


**Fig. S34** (**a**) Phase angle and (**b**) total impedance as a function of frequency for CNF-CCF-A and CCF-A under different states (30% compression and 80% tension), obtained from impedance spectroscopy measurements


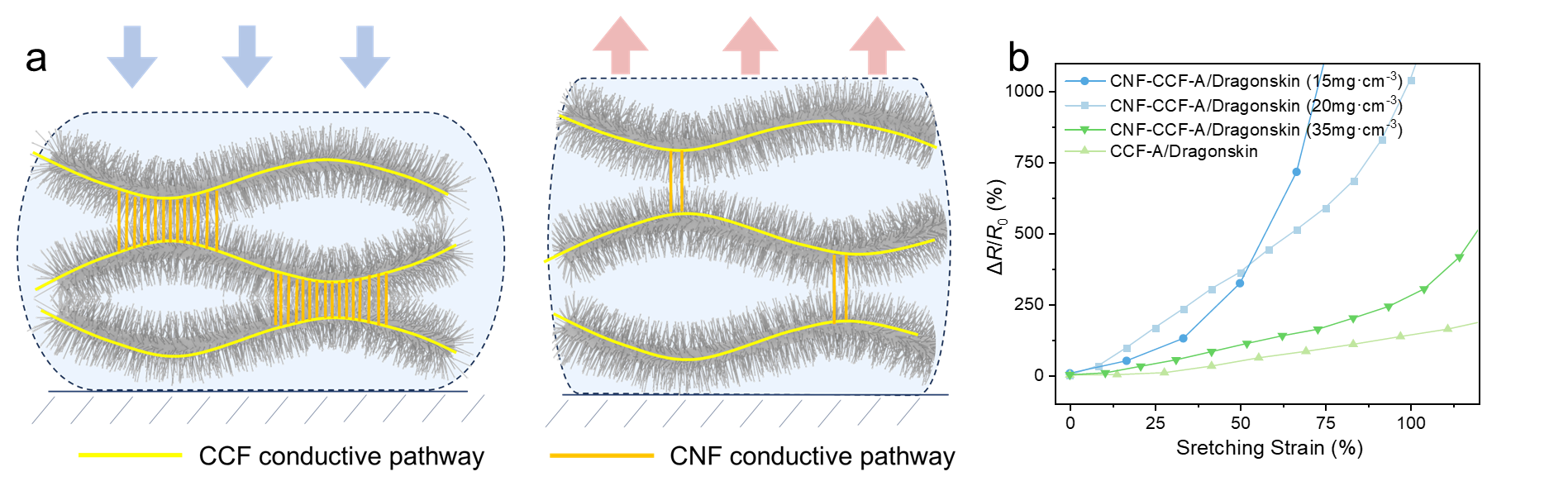


**Fig. S35** (**a**) Schematic illustration of the differences in conductive pathway evolution in a heterogeneous conductive structure under tensile and compressive deformation. (**b**) Electromechanical response signals of aerogels with different densities under tensile strain


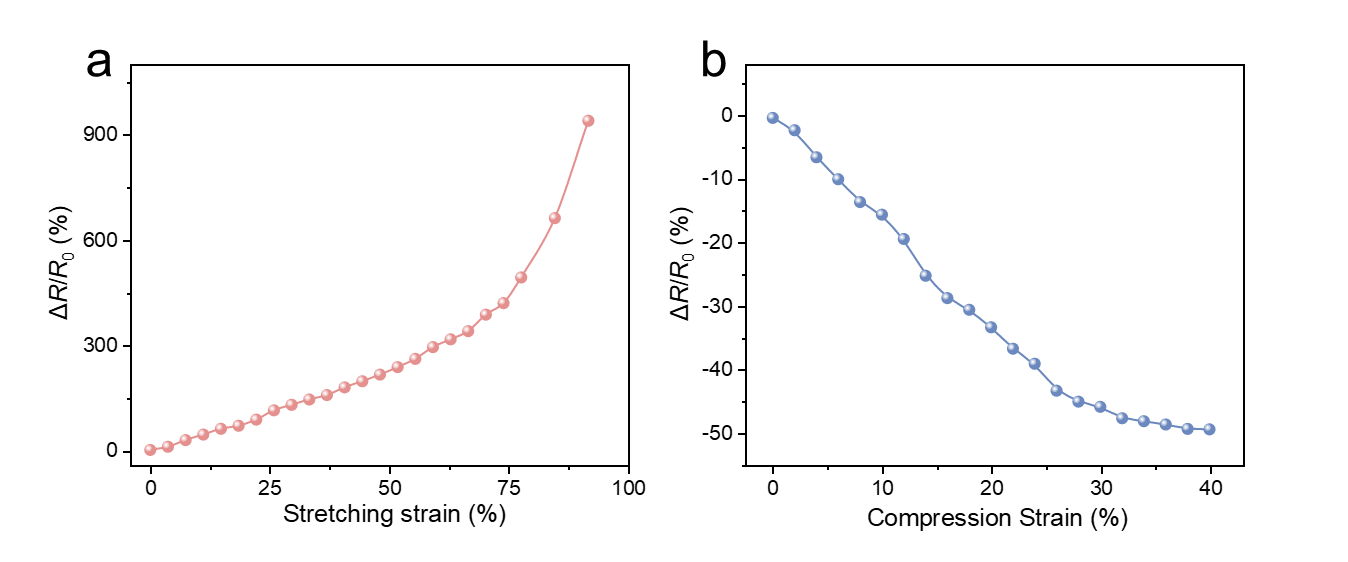


**Fig. S36** (**a**) Electrical response of the CNFs-CCFs-A/Dragonskin device fabricated using a black waste plastic packaging box as the carbon feedstock under tensile loading, and (**b**) under compressive loading


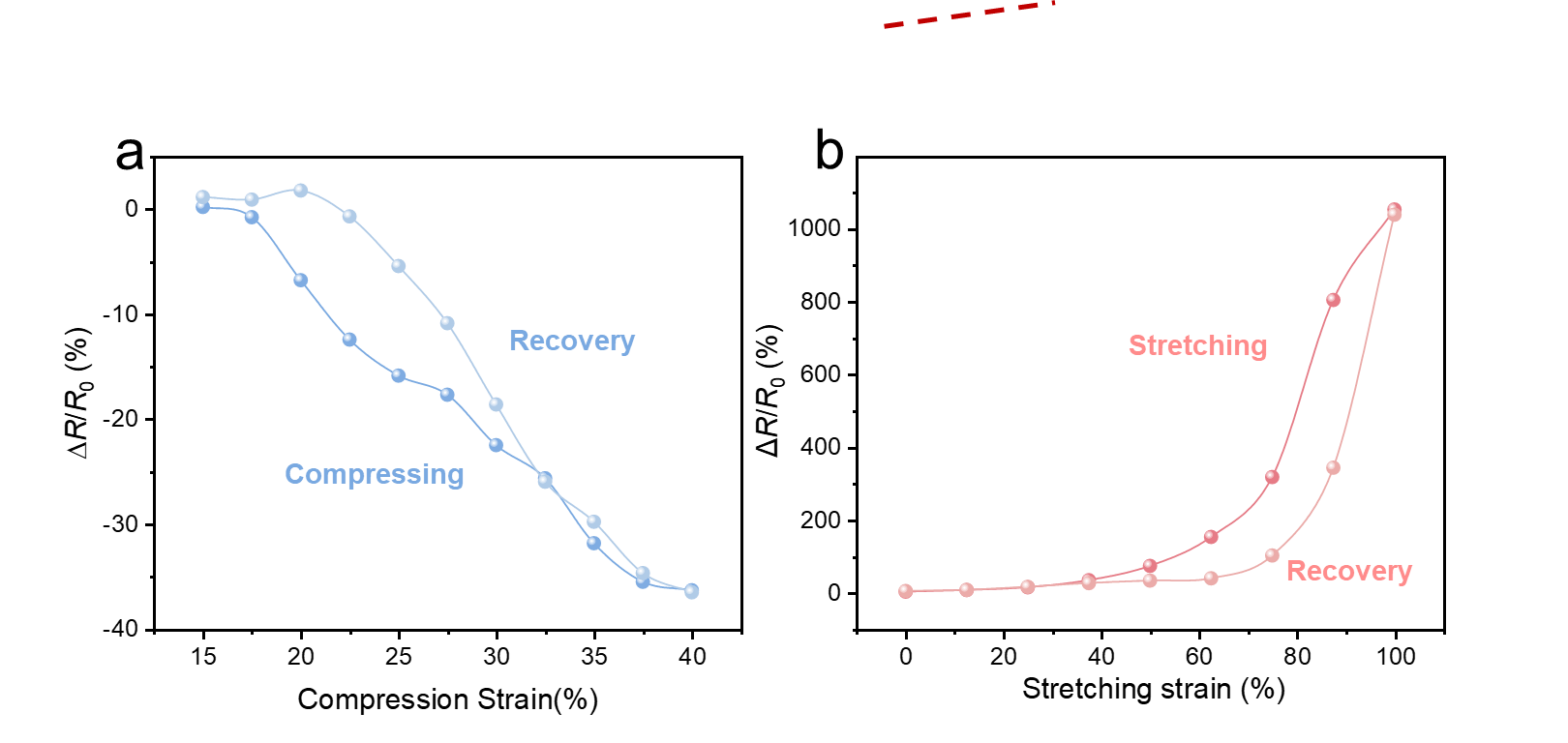


**Fig. S37 (a)** Compression-recovery response curve under 0-40% compressive strain and **(b)** tensile-recovery response curve under 0-100% tensile strain of the Ni-CNFs-CCFs-A/Dragonskin composite


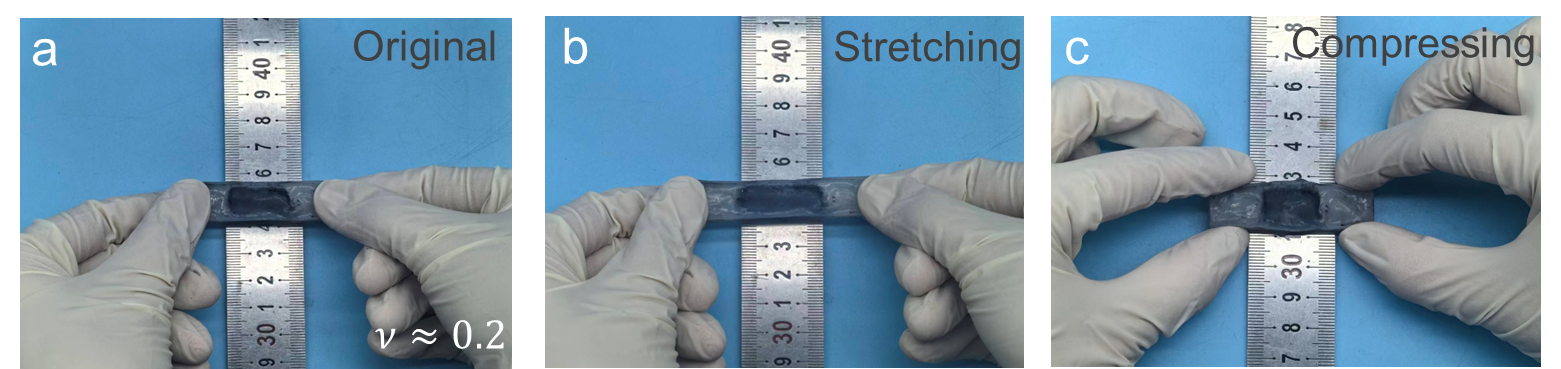


**Fig. S38** (**a**) Optical photograph of the sensor in the original state. (**b**)Optical photographs showing the Poisson effect during stretching and (**c**) compressing


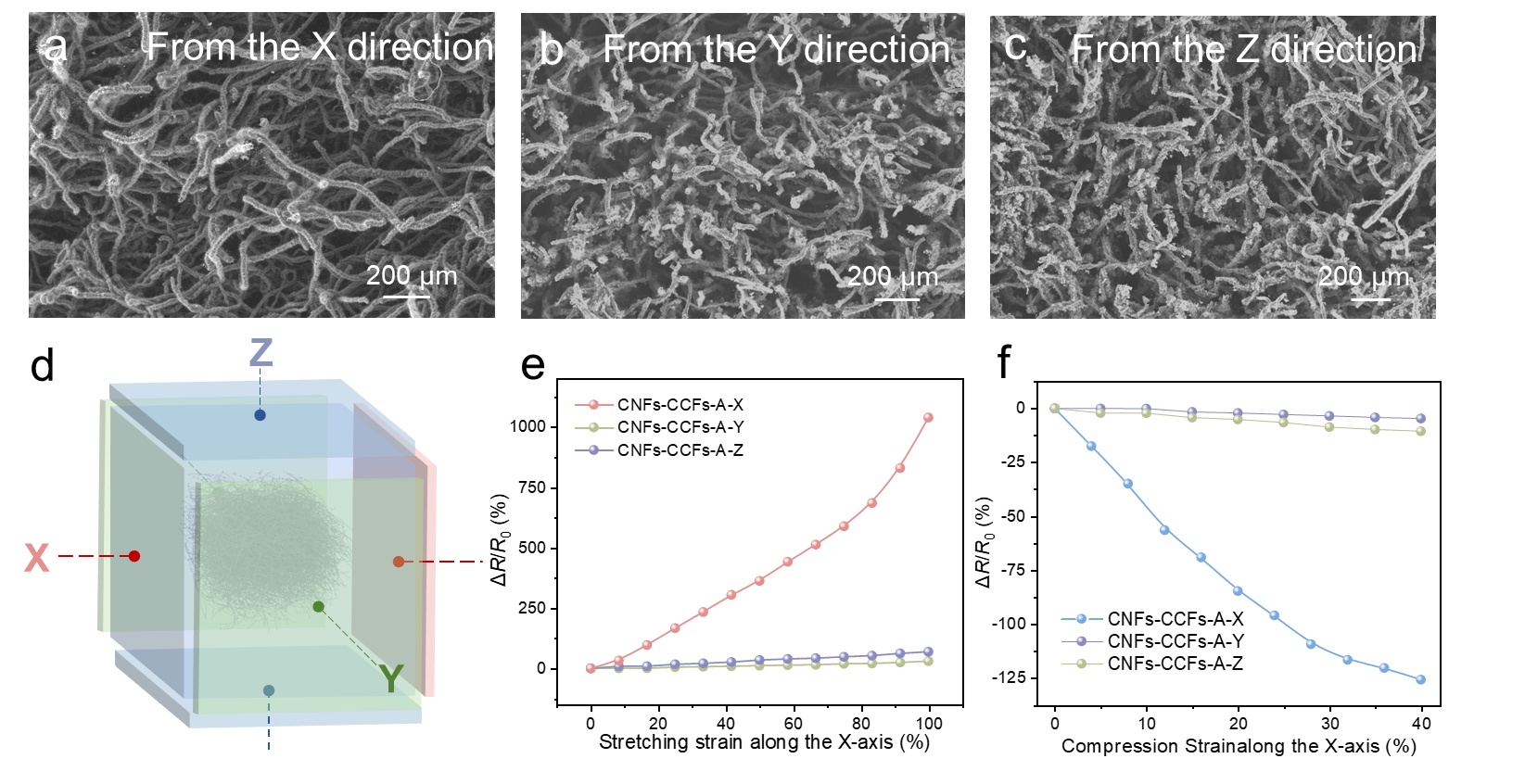


**Fig. S39** SEM images of cross-sections of the CCF scaffold after vertical cutting along the (**a**) X, (**b**) Y, and (**c**) Z directions. (**d**) Schematic illustration of XYZ-directional decoupling of the CNFs-CCFs-A/Dragon Skin device. (**e**) Relative resistance variation (Δ𝑅/𝑅_0_) measured along the three axes during stretching and (**f**) compression along the X direction


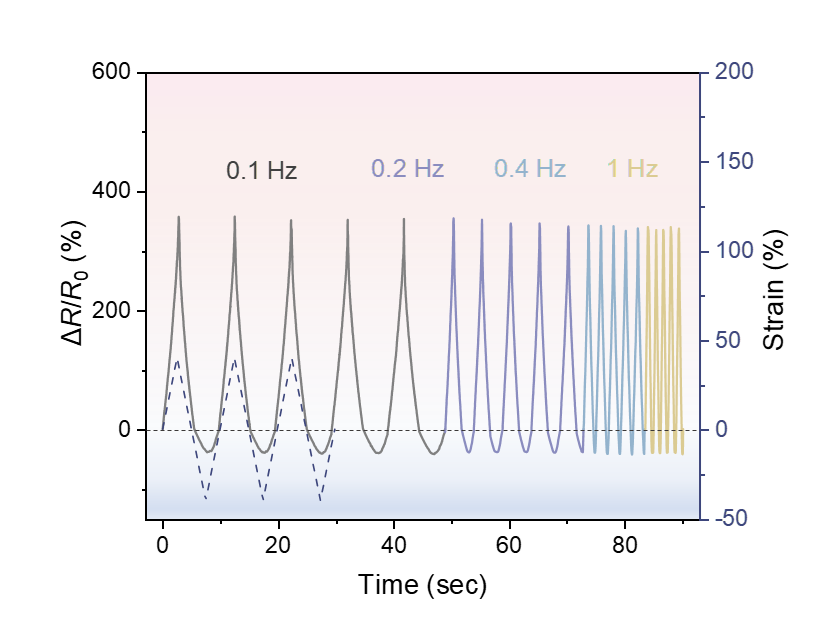


**Fig. S40** Signal reliability under alternating tensile-compressive loading at 40% strain across frequencies from 0.1 to 1 Hz


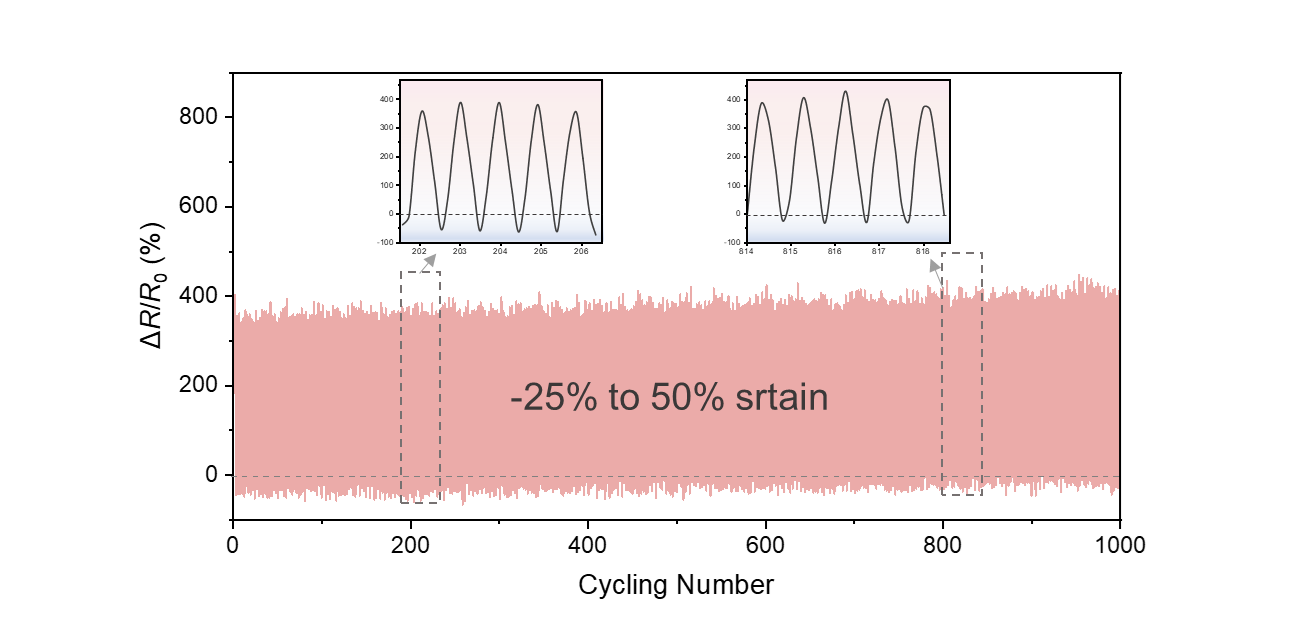


**Fig. S41** Durability test over 1000 cycles between +50% tensile and −25% compressive strain


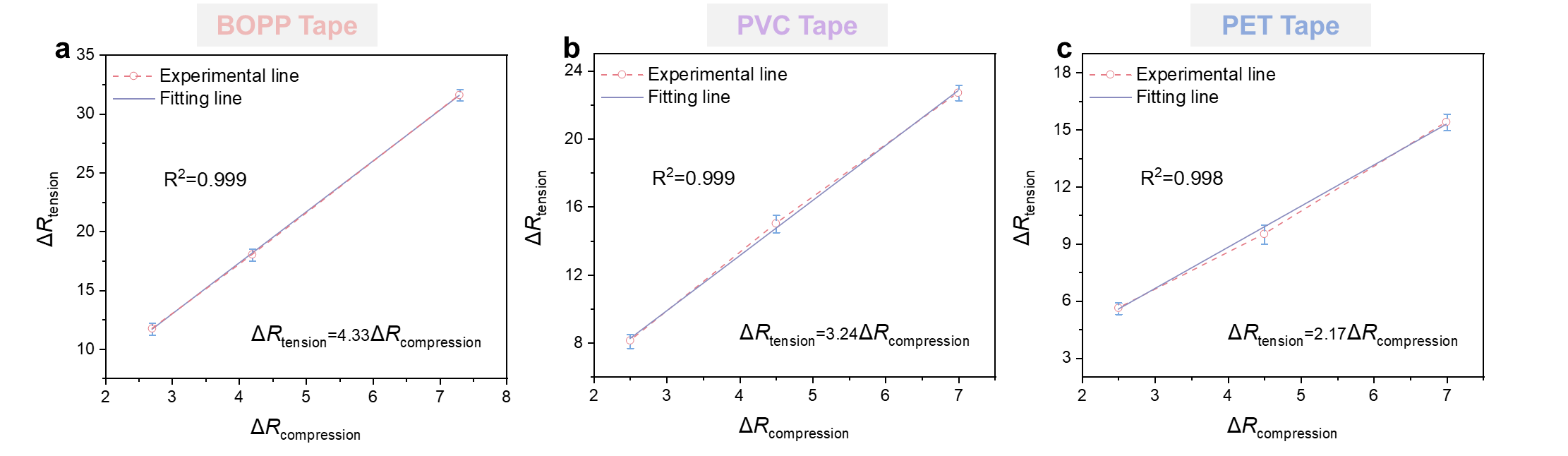


**Fig. S42** (**a**) BOPP tape, (**b**) PVC tape, and (**c**) PET tape were used to calibrate interfacial adhesion based on the ratio of electrical signals generated after applying force to their surfaces, where the ratio *k* follows the empirical relationship Δ*R*_tension_ = *k*·Δ*R*_compression_, with *k* being a constant


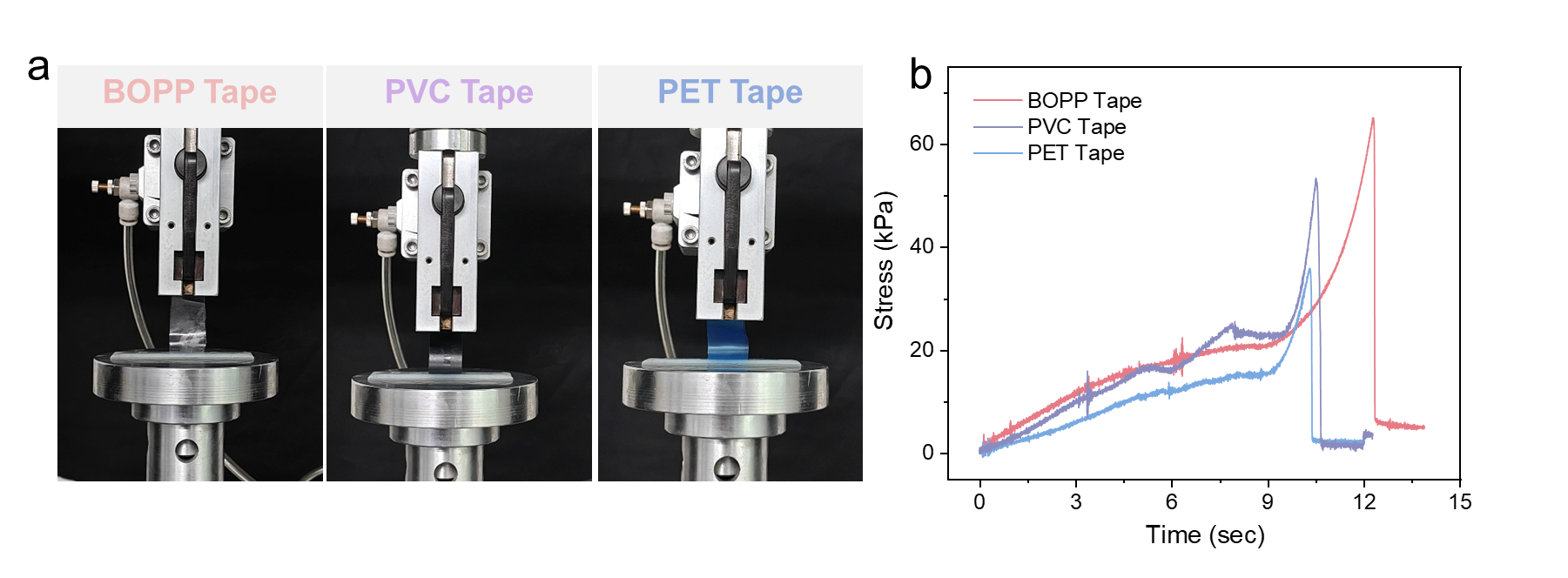


**Fig. S43 (a)** Mechanical adhesion tests performed on different adhesive tapes with the same contact area and **(b)** the corresponding stress–strain curves


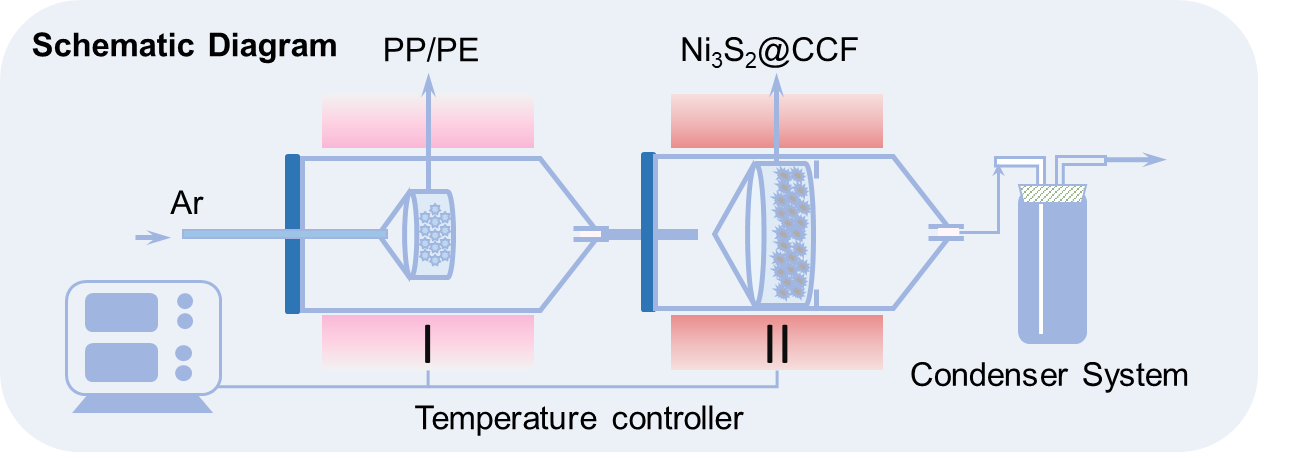


**Fig. S44** Schematic illustration of the dual-zone experimental setup used for the preparation of CNFs-CCFs-A, in which Zone I is employed for the pyrolysis of plastic precursors, and Zone II is used for the growth of carbon nanofibers

**Table S1** Comparison of CNF diameter and length parameters between Ni-S_x_-CNFs and Ni-CNFs

|  | Ni-S_x_-CNFs | Ni-CNFs |
| --- | --- | --- |
| D_max_ nm | 590 | 600 |
| D_min_ nm | 170 | 240 |
| D_avg_ nm | **280** | **440** |
| L_max_ μm | 20.03 | 29.23 |
| L_min_ μm | 7.98 | 1.91 |
| L_avg_ μm | **15.13** | **8.61** |

**Table S2** Performance comparison of CNFs-CCFs-A sensors with previously reported devices

| **Materials** | **Tension_max_**  **(%)** | **GF_tension_**  **(linear region)** | **Compression_max_**  **(%)** | **GF_compression_**  **(linear region)** | **Sensing mode** | **Refs.** |
| --- | --- | --- | --- | --- | --- | --- |
| **BCNF/MTMS/rGO** | / | / | 50 | 4.97 at 10% | / | [S1] |
| **PI/G aerogel** | / | / | 60 | 0.96 | / | [S2] |
| **MWCNT@Ecoflex** | 105 | 10 | / | / | / | [S3] |
| **MXene/BC-TPU** | 80 | 1.46 at 40% | 80 | 1.87 at 30% | Heteroaxial strain | [S4] |
| **Molybdenum**  **Carbide-graphene** | 25 | 73 at 0.25% | 10 | 43 at 0.2% | Heteroaxial strain | [S5] |
| **Carbon black** | 300 | 13.2 at 25% | 45 | 0.88 at 45% | Heteroaxial strain | [S6] |
| **Carbon nanotubes** | 250 | 9.4 at 30% | 50 | 1.18 at 50% | Heteroaxial strain | [S7] |
| **Reduced graphene oxide** | 50 | 1.39 at 16% | 50 | 1.74 | Heteroaxial strain | [S8] |
| **Porous graphene–carbon composite** | 60 | 3 at 20% | 60 | 1.75 at 20% | Coaxial strain | [S9] |
| **Graphene and MWCNTs** | 100 | 0.1 | 14 | 5.7 | Coaxial strain | [S10] |
| **AgNW@PU** | 60 | 2.19 at 35% | 60 | 1.45 at 60% | Coaxial strain | [S11] |
| **CNOs/CNTs-SEBS-PU** | 120 | 9.8 at 2% | 80 | 0.08 at 28% | Coaxial strain | [S12] |
| **CNFs-CCFs-A** | **100%** | **7.8 at 78%** | **40%** | **1.7 at 28%** | **Coaxial strain** | **This work** |

*Engineering strain was adopted consistently, and the GF was extracted from the equivalent initial linear strain regime (based on linear fitting).

**Supplementary References**

1. H. Cai, J. Zheng, J. Zhang, W. Zhang, Y. Zhao et al., Synergistically Reinforced Bamboo Cellulose–Graphene Aerogel Sensors with Highly Elasticity and Strain Sensitivity. ACS Appl. Electron. Mater. **7**(16), 7755–7765 (2025). <https://doi.org/10.1021/acsaelm.5c01129>
2. S. Zheng, L. Jiang, F. Chang, C. Zhang, N. Ma et al., Preparation of robust and light-weight anisotropic polyimide/graphene composite aerogels for strain sensors. Polym. Adv. Technol. **35**(1), e6231 (2024). <https://doi.org/10.1002/pat.6231>
3. Z. Guo, X. Hu, Y. Chen, Y. Ma, F. Zhao et al., Soft, Stretchable, High-Sensitivity, Multi-Walled Carbon Nanotube-Based Strain Sensor for Joint Healthcare. Nanomaterials **15**(5), 332 (2025). <https://doi.org/10.3390/nano15050332>
4. C. Bai, S. Jia, W. Chen, L. Li, Y. Zhang et al., Dual-Network MXene/Polyurethane Composite Foams for Both Stretchable and Compressible Electromagnetic Interference Shielding and Strain Sensors. ACS Appl. Mater. Interfaces **17**(7), 11108–11116 (2025). [https://doi.org/10.1021/acsami.4c21321](%20https:/doi.org/10.1021/acsami.4c21321)
5. Y. Long, P. He, R. Xu, T. Hayasaka, Z. Shao et al., Molybdenum-carbide-graphene composites for paper-based strain and acoustic pressure sensors. Carbon **157**, 594–601 (2020). <https://doi.org/10.1016/j.carbon.2019.10.083>
6. T. Gong, J. Jia, X.-R. Sun, W.-D. Li, K. Ke et al., Design strategy for hierarchical structure of carbon black on microporous elastomer surface toward stretchable and compressive strain sensors. Carbon **206**, 53–61 (2023). <https://doi.org/10.1016/j.carbon.2023.02.008>
7. X. Guo, T. Xing, J. Feng, Simultaneously Stretchable and Compressible Flexible Strain Sensors Based on Carbon Nanotube Composites for Motion Monitoring and Human–Computer Interactions. ACS Appl. Nano Mater. **5**(12), 18427–18437 (2022). <https://doi.org/10.1021/acsanm.2c04267>
8. L. Cheng, J. Feng, Facile fabrication of stretchable and compressible strain sensors by coating and integrating low-cost melamine foam scaffolds with reduced graphene oxide and poly (styrene-b-ethylene-butylene-b-styrene). Chem. Eng. J. **398**, 125429 (2020). <https://doi.org/10.1016/j.cej.2020.125429>
9. H.-L. Gao, Z.-Y. Wang, C. Cui, J.-Z. Bao, Y.-B. Zhu et al., A Highly Compressible and Stretchable Carbon Spring for Smart Vibration and Magnetism Sensors. Adv. Mater. **33**(39), 2102724 (2021). <https://doi.org/10.1002/adma.202102724>
10. F. Guo, Y. Jiang, Z. Xu, Y. Xiao, B. Fang et al., Highly stretchable carbon aerogels. Nat. Commun. **9**(1), 881 (2018). <https://doi.org/10.1038/s41467-018-03268-y>
11. M. F. Ahmed, Y. Li, C. Zeng, Stretchable and compressible piezoresistive sensors from auxetic foam and silver nanowire. Mater. Chem. Phys. **229**, 167–173 (2019). <https://doi.org/10.1016/j.matchemphys.2019.03.015>
12. S. S. Nemala, B. Bernardino, R. M. R. Pinto, V. Lopes, P. Alpuim et al., Electrically Conductive Nanocarbon/Elastomer Composite Inks for Flexible and Wearable Strain Sensing. Small **21**(50), e06844 (2025). <https://doi.org/10.1002/smll.202506844>
